# Impacts of Climate Change and its perception in communities surrounding Lake Titicaca, Peru-Bolivia.

**DOI:** 10.12688/f1000research.176641.1

**Published:** 2026-03-19

**Authors:** Alberto Enrique Garcia Rivero, Yuri Vladimir Sandoval Montes, Edwin Natividad Gabriel Campos, Javier Alfredo Núñez Villalba, Zaniel Israel Novoa Goicochea

**Affiliations:** 1 Universidad Nacional Mayor de San Marcos Facultad de Ciencias Sociales, Lima District, Lima Region, Peru; 2 Universidad Mayor de San Andres Facultad de Ingenieria, La Paz, Bolivia; 3 Pontificia Universidad Catóica del Perú Departamento de Humanidades, San Miguel, Lima, Peru

**Keywords:** climate change; drought; precipitation; fishery; agriculture; adaptation, mitigation; Lake Titicaca.

## Abstract

**Background:**

Lake Titicaca underpins the livelihoods, culture, and ecosystems of high Andean Aymara and lakeside communities in Peru and Bolivia. Recent multi-year declines in lake level, rising temperatures, and greater climate variability threaten water supply, agriculture, fisheries, tourism, and public health. This study characterizes climate change impacts and local perceptions in lakeside sectors to inform adaptation and cross-border management.

**Methods:**

A multidisciplinary, participatory approach combined: (1) exhaustive document review of physical and cultural drivers; (2) fieldwork (November 2024 and June 2025) in Quehuaya (Cohana Bay, Bolivia), Capachica (Puno Bay, Peru), Guaqui (Bolivia), and Puno urban area; and (3) participatory methods including community workshops with social mapping and surveys, six in-depth interviews, and a MINCETUR ecotourism seminar survey (n = 26). Data were triangulated with institutional meetings and synthesized quantitatively.

**Results:**

Eighty-five percent of respondents reported observed climate change indicators; seventy-four percent noted decreased rainfall, and fifty-eight percent reported hotter days. Sectoral impacts included agriculture (61% affected; reduced yields and altered planting calendars), fishing (52% decline in species or volumes), livestock (50% impacts on pasture and water; 42% increased disease), and tourism (27–29% affected by reduced navigability and pollution). Fifty-three percent reported local adaptation actions (e.g., tolerant crops, water reservoirs, traditional practices), while external support from government or NGOs was limited (~18–19%). Primary needs were financing (76%), improved climate information (52%), and organizational strengthening (26%). Participatory mapping documented shoreline retreat and cultivation of exposed lakebeds. High perceived urgency and reported migration increases (53%) highlight socioeconomic vulnerability.

**Conclusions:**

Communities around Lake Titicaca face a chronic water-deficit syndrome driven by climatic and local stressors, producing cascading ecological, economic, health, and cultural impacts. Reactive localized adaptations exist but are insufficient. Effective response requires integrated binational strategies: improved water storage and irrigation, wetland restoration, pollution control, real-time climate information, targeted financing, and strengthened institutional coordination.

**Abbreviations**
ALT: Binational Authority of Lake Titicaca
CONCYTEC: National Council of Science, Technology and Innovation of Peru
IIGEO-UMSA
: Institute of Geographic Research
MINCETUR: Ministry of Foreign Trade and Tourism of Peru
PROCIENCIA: National Program for Scientific Research and Advanced Studies

## 1. Introduction

Climate change has emerged as one of the main factors transforming ecosystems and altering socio-environmental systems, affecting both nature and human communities. Recent research (2020–2024) evidences various impacts, such as ecological degradation and socio-environmental vulnerability, linked to ecosystem alterations and a decline in ecosystem services. ^1– 5^ In socially exposed contexts, climate risk exacerbates economic and social inequalities, increasing the vulnerability of populations dependent on natural resources. ^6– 9^

Climate variability, which affects precipitation patterns, temperature, and water balances, stands out as a critical dimension of climate change, necessitating predictive models and adaptation strategies based on hydrometeorological evidence. ^10– 13^ It also leads to alterations in biodiversity, characterized by changes in species distribution and abundance, where a loss of ecological functionality can also be observed. ^14– 16^

A review of studies from the last five years confirms that the effects of climate change manifest in multiple dimensions, including climate variability, ecological degradation, habitat loss, and increased socio-environmental vulnerability. ^17– 30^ These impacts transcend ecological concerns, affecting social and territorial structures and demanding integrated approaches to environmental management and territorial planning.

Lake Titicaca, the center of a vast ecological, economic, and cultural network in the high plateau region, has faced a marked and concerning decline in water levels for years. This phenomenon, explained by a combination of global and regional climatic factors alongside local resource management practices, poses a structural threat to the lakeside communities that depend on the lake for water, food, income, and cultural significance. The current research, funded by the PROCIENCIA program of CONCYTEC (Peru) in 2024, aims to identify and characterize the main impacts of climate change on the populations inhabiting the lakeside sector of Titicaca, focusing on the Bay of Cohana (Bolivia) and the Bay of Puno (Peru), and to provide elements to guide policies for adaptation and integrated cross-border management.

The Aymara communities and other lakeside populations maintain a close, multisectoral relationship with the lake: artisanal fishing and small-scale agriculture form the basis of food security and economic sustenance; lake water is used for human consumption, irrigation, and productive activities; and the landscapes and practices associated with the lake support a cultural heritage that includes traditional knowledge, rituals, and community organization. The sustained reduction in water volume and variability—particularly the recurrence of drought episodes—alters local hydrological regimes, deteriorates the productivity of agricultural and fishing systems, and jeopardizes the provision of environmental and tourism services. ^31– 34^

To address this complexity, the research aimed to determine the impacts of climate change on lakeside communities around Lake Titicaca, adopting a multidimensional approach that combined a documentary review of studies on the physical, socioeconomic, historical, and cultural environment; participatory fieldwork in representative communities of the bays of Cohana and Puno (community workshops developing participatory social mapping, surveys, semi-structured interviews with residents and local authorities, and dialogues with institutional actors).

The multifaceted effects identified at the community level include: a decrease in crop production and variety due to reduced irrigation water availability; loss of biomass and fishing habitats affecting fish catch and quality; degradation of wetlands and lakeshore areas that reduce water regulation capacity and biodiversity ^34, 35^; and an increase in concentrated pollutants due to lower water volumes, with implications for public health. ^36^ These productive and health impacts are accompanied by profound sociocultural consequences: a breakdown of traditional collective water management practices, erosion of knowledge related to fishing and agriculture, and disruption of rituals linked to the lacustrine cycle. Finally, the deterioration of livelihoods drives internal and cross-border migration processes that reconfigure demographic, economic, and family dynamics, creating additional vulnerabilities for communities remaining in the territory.

## 2. Materials and methods

To comprehensively address the perception of climate change impacts on lakeside communities around Lake Titicaca, this research adopted a multidisciplinary and participatory approach, integrating both qualitative and quantitative techniques. The methodology was designed in alignment with the objectives of identifying the main impacts on selected communities from the bays of Cohana (Bolivia) and Puno (Peru), along with other areas of interest (see Figure 1), and to provide elements that can guide adaptation and mitigation policies in response to climate change impacts.

**Figure 1.**
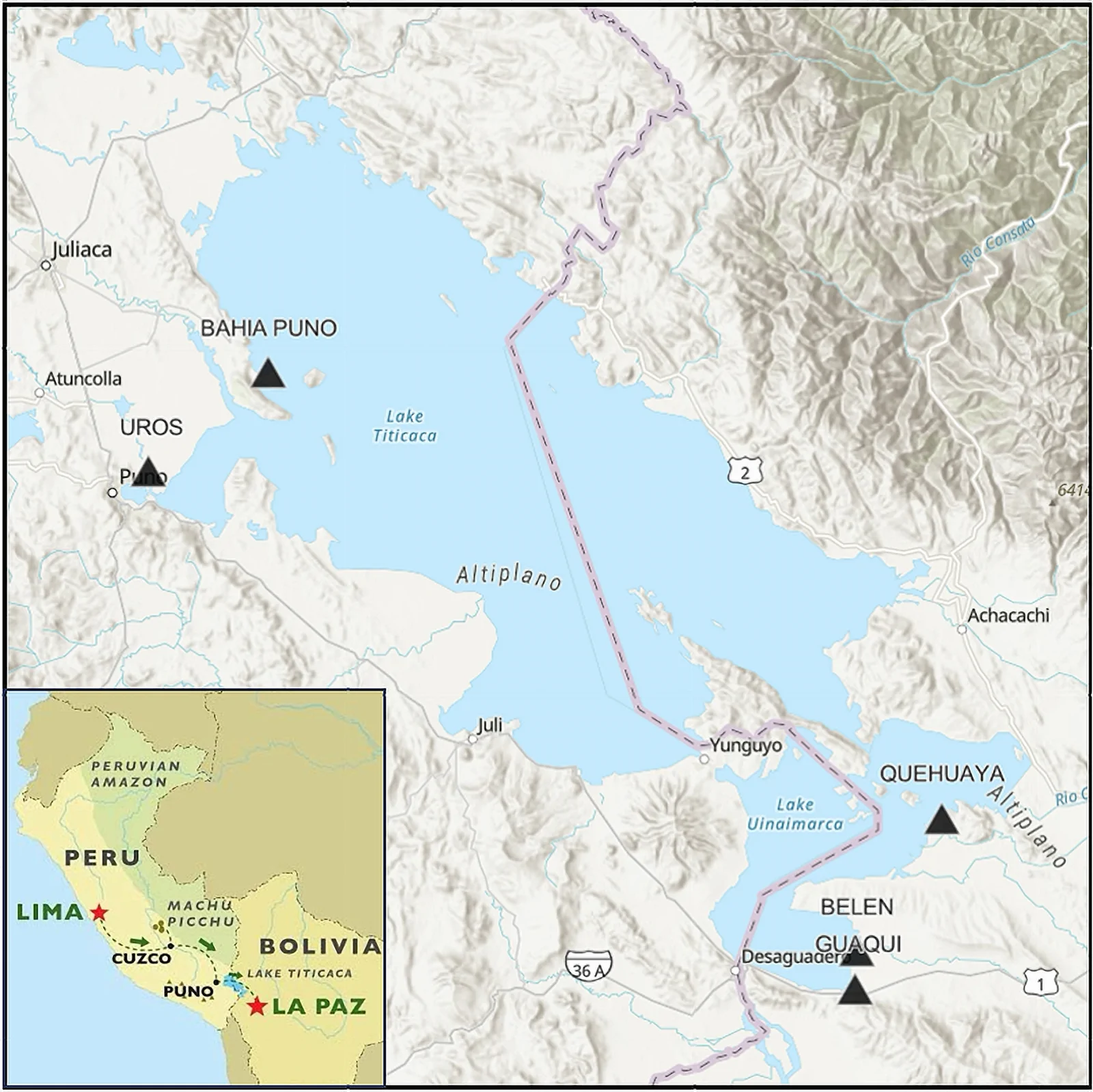
Location of the study areas for this research.

The development of this work was structured into three fundamental phases, described below:

First Phase: Document Review

An exhaustive review of scientific literature, institutional reports, and historical studies related to the physical, socio-economic, and cultural environment of Lake Titicaca and its communities was conducted, along with an analysis of climate change, climate variability, and their main impacts. This review allowed for contextualizing the observed changes in the lake system, understanding long-term trends, and identifying factors contributing to modifications in hydrological and social regimes.

Second Phase: Fieldwork

Fieldwork was conducted in two periods: in November 2024 (at the end of the dry season) and in June 2025 (at the beginning of the dry season). The fieldwork took place in the communities of Quehuaya in the Bay of Cohana and Capachica in the Bay of Puno, chosen for their representativeness and vulnerability to changes in the lake. Additionally, work was done in the Bolivian sector in Guaqui, a locality and municipality in the Ingavi province within the La Paz department, located on the shores of Lake Titicaca. In the Peruvian area, the study focused on an area of the city of Puno where the main sewage drains flow into the lake.

The activities included participatory workshops with residents to gather traditional knowledge, perceptions of changes in the lake, and existing adaptation strategies. This involved working with community members in strict compliance with the “Code of Ethics in Research of the National University of San Marcos,” approved by Rectoral Resolution No. 012648–2023-R/UNMSM of November 21, 2023 ( https://letras.unmsm.edu.pe/wp-content/uploads/2023/11/2023_Codigo-de-etica-del-investigador.pdf).

For this study, informed verbal consent was obtained from the communities where the research was conducted. In these communities, consent is traditionally given verbally; it was obtained after presenting, explaining, and reaching an agreement with community leaders on the purpose of the project, the main activities, and the expected benefits before starting the project, through a preliminary visit to the study area. The communities’ tacit and practical confirmation of that consent was evidenced by the participation of their members and leaders in the various workshops and field visits in the study area. In summary, the only valid reason for using verbal informed consent is that the communities in this study do not customarily provide written, signed consent; oral communication and personal word carry greater weight in these populations.

Surveys were applied to all workshop participants (see Appendix A), and non-structured interviews were conducted with community leaders, farmers, fishermen, and local authorities to identify the most relevant threats and community responses to climate alterations and their consequences on ecosystems and inhabitants.

Participatory social mapping was developed during the workshops, viewing the map as a dynamic social message, a “collectivized film” in constant evolution, moving away from positivism while prioritizing dialogue and lived experiences. ^37, 38^ This approach operates on the principle that those who inhabit a territory know it best ^39^ and gives voice to local knowledge through accessible expressions like drawings and iconography, ^40^ promoting self-recognition and the strengthening of sociocultural heritage. ^39^ It is especially useful for communities vulnerable to climate change as it incorporates local knowledge in identifying issues and formulating mitigation and adaptation strategies, facilitates the visualization of changes, encourages participation in decision-making, fosters the creation of collective plans, and enhances communication and community commitment in climate research. ^41– 44^

Additionally, we attended a training seminar organized by MINCETUR, which convened a significant group of residents from the surrounding lake communities actively engaged in ecotourism. At the conclusion of the seminar, participants were asked to complete a survey (refer to Appendix B).

Furthermore, we conducted meetings with governmental institutions, NGOs, and water resource management organizations to gain insights into existing policies and explore opportunities for enhanced coordination.

Third Phase: Data Processing and Analysis

The data collected during the workshops, seminars, and field visits were carefully processed to identify patterns and emerging categories related to impacts and adaptation strategies. Ultimately, all information was systematically integrated and interpreted, resulting in the findings and conclusions presented in this article.

## 3. Results

Below is a summary of the main results obtained and their interpretation.

### 3.1 Community workshops in Quehuaya and Capachica

In November 2024, two participatory community workshops were held—one in the Cohana Bay Sector of Quehuaya district, La Paz, Bolivia, and the other in the Puno Bay Sector of Capachica district, Puno, Peru. Additionally, participation took place in the seminar-workshop organized by MINCETUR in Puno, which involved key promoters of ecotourism and experiential tourism around Lake Titicaca and its surroundings.

3.1.1 Surveys

The main results from the survey conducted among 62 residents of the studied communities are summarized in Appendix C-Table C1.1, categorized by gender. The sample consisted of 33 women and 29 men, all over the age of 16, with an average age of 48. Regarding education, 66% of the respondents have not exceeded primary education, 24% have completed secondary education, and only 13% have pursued technical or university studies.

Among the group, 66% are engaged in agriculture, 44% in livestock, 26% in fishing, as well as tourism activities, while the rest are involved in crafts and other activities. It’s important to note that many participants practice more than one activity, such as agriculture, livestock, and fishing.

An impressive 85% identified various indicators of climate change impacts, highlighting a decrease in rainfall (74%), hotter days (58%), increased cold and snow (29%), and heavier or irregular rains. They also acknowledge that these changes have affected agriculture (61%), fishing (31%), tourism (29%), and livestock (24%) significantly. In agriculture, the main impacts include reduced production of various crops (56%), such as potatoes, quinoa, broad beans, and valde, as well as the need to change traditional planting calendars (48%).

Livestock has also been affected, with impacts on pasture areas and water availability (50%), an increase in livestock diseases (42%), and consequently a decrease in the number of livestock (40%). For the fishing sector, a decrease in fish availability regarding species and volumes has been reported (52%), especially affecting species like carachi, pejerrey, umanto, ispi, suche, and boga. Some fishers have reported needing to move to areas with better availability within Lake Titicaca.

The tourism sector, like the previous ones, shows significant impacts acknowledged by 27% of the surveyed individuals. Among these impacts, the loss of lake water stands out, which primarily hinders navigation and communication on Lake Titicaca, jeopardizing excursions and experiential activities included in the region’s tourism offerings. Additionally, alarming levels of lake pollution and loss of water volume threaten the survival of this important aquatic ecosystem. When compounded by severe issues often caused by social protests, tourism activities are considerably affected.

In response to these direct and indirect impacts of climate change, the communities are developing adaptation and/or mitigation measures. In the case of the studied communities, 53% of surveyed residents report implementing some measures, such as introducing new climate-resistant crop varieties (23%), constructing water reservoirs, using traditional practices mainly in agriculture and livestock (19%), participating in climate change training (18%), diversifying activities like tourism and crafts (15%), among others.

Moreover, in response to this crisis, many community members have started cultivating Andean products along the lake’s shores, taking advantage of soil moisture and the space created by receding waters. This illustrates their resilience in the face of drought and underscores the community’s adaptation to new living conditions ( Figure 2).

**Figure 2.**
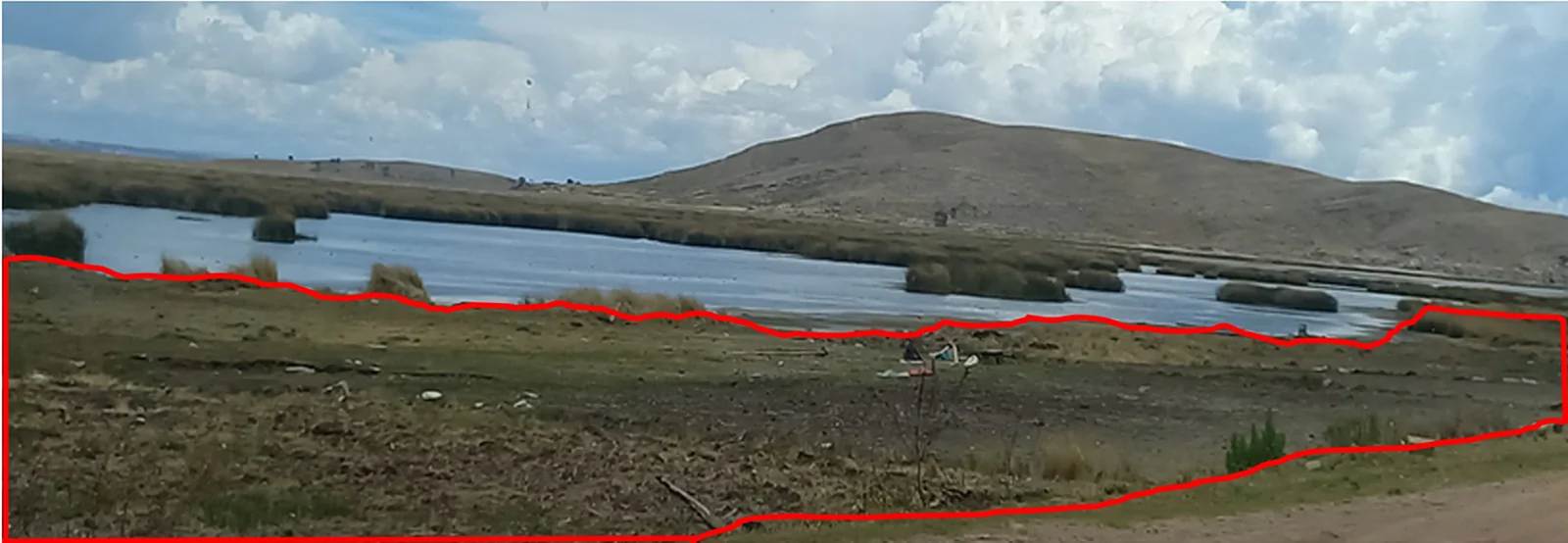
Example of a section of the lake that has become exposed due to decreasing water levels and is being utilized for the development of new crops.

In the development of these activities to combat climate change, communities have received support from various entities and organizations. This study shows that government entities and NGOs contribute almost equally (18% and 19%, respectively), followed by the National Lake Authority (16%) and universities (13%).

Lastly, respondents expressed the main needs of the communities to better address climate change. The most significant need, highlighted by 76% of participants, is for funds or loans to develop various tasks aimed at improving infrastructure resilience, building new water reservoirs, and creating more efficient irrigation systems, among others. Similarly, 52% called for improved and more comprehensive information about climate issues, which would enhance their preparedness for different weather phenomena. Additionally, 26% emphasized the importance of organizational strengthening, both within communities and in their relationships with various levels of government, civil society, academia, and the private sector.

A significant 87% of respondents consider climate change a critical issue, recognizing its effects on 63% of agricultural activities. Additionally, 53% report increased migration, particularly among young men, which poses a direct threat to community survival. Furthermore, 47% emphasize the health problems associated with climate change, 40% cite its impacts on livestock, while a smaller portion, 24%, notes its effects on tourism, and 15% on crafts.

When analyzing the survey results, based on the opinions of the groups by economic activity (Appendix C—Table C2.1), interesting information emerges that is summarized below according to each of the main topics.

The characteristics of the interviewees reveal an average age of 48 years for the Fishing group, 35 years for the crafts group, and 36 years for Tourism, which are notably younger than the Agriculture and Livestock groups, both at 55 years. This suggests a possible generational difference in participation in these activities. The crafts activity is predominantly male (80%), with only 20% female participation, while the tourism sector shows perfect gender balance (50% women, 50% men). The Agriculture, Livestock, and Fishing groups have a slight male majority but significant female participation (between 41% and 46%). In terms of education levels, the crafts and Tourism groups have the highest levels, with no community members lacking education or only having completed primary school. Secondary education (40–44%) and technical training (38–60%) are the most common. This aligns with their lower average age. Agriculture and Livestock members primarily have primary education (56–59%), followed by secondary education, while the Fishing group has an intermediate educational level, with primary education being the most common (44%), but also significant participation in secondary (25%) and technical education (31%).

There is a high diversification of productive activities across nearly all groups, indicating that community members do not engage exclusively in one activity. The Fishing, crafts, and Tourism groups display the greatest diversification, with a high percentage of members involved in multiple activities (e.g., fishermen also engaging in agriculture and livestock). Notably, 100% of community members in both the Livestock and Fishing groups also participate in Agriculture, suggesting a strong interdependence or combination of these activities.

Regarding perceptions of Climate Change, there is an overwhelming consensus on the occurrence of such changes in the last 10–20 years. The crafts (100%), Tourism (94%), and Fishing (88%) groups perceive these changes most broadly, although Agriculture (78%) and Livestock (74%) also show a high perception.

The perception that climate change impacts productive activities is very high across all groups. The crafts (100%) and Tourism (94%) groups report the most widespread impacts, with each group tending to report the greatest effect on their main activity (e.g., 75% of the Tourism group reports impacts on tourism). However, there is also notable cross-impact: agriculture is frequently mentioned as affected even by non-agricultural groups (e.g., 81% of fishermen perceive impacts on agriculture).

When analyzing the impacts on each main activity, the effects on potato, quinoa, and broad bean crops are the most consistently reported by community members across all groups. For the Fishing activity, the Fishing group reports 100% impact on various species, with carachi (100%) being the most affected, followed by pejerey (81%) and umanto (75%). Other activities are perceived to have impacts on these species, with carachi being the most mentioned by everyone.

Other activities, like Crafts are only perceived as highly impacted by their own group. The other groups report a significantly lower perceived impact. In terms of tourism, impacts are strongly felt within the Tourism (94%) and crafts (80%) groups, with the main causes of impact being lake water loss (50%) and water pollution (50%), followed by strikes due to social protests (38%).

Regarding adaptation strategies, a significant portion of all groups (between 40% and 74%) has implemented adaptation measures. The use of new climate-resistant crop varieties, the construction of water reservoirs, and the adoption of traditional practices are the most common. Overall, support for adaptation is relatively low, with an average of 25–38% of community members receiving it, and the Fishing and Tourism groups receiving the most (38% each), with the main sources of support being the government, NGOs, and for the Tourism group, also universities. The vast majority of community members (between 60% and 81%) perceive the need to take action to address climate change, with the Tourism group feeling the strongest urgency (81%). There is a general emphasis on the need for funding/loans and timely, accurate weather information.

There is a near-universal consensus (between 80% and 94%) when considering climate change as a major problem, particularly among the Tourism (94%) and Fishing (88%) groups. Reasons vary but center around direct impacts on their livelihoods and broader social issues, with migration being a significant concern for several groups—especially the main reason for the Tourism group (75%) and very relevant for crafts (60%), Livestock (59%), and Agriculture (51%). Public health is a major concern for the crafts group (80%) and also significant for the Tourism group (56%).

There is a general trend where each group prioritizes the impact on their main activity (e.g., crops for Agriculture, fishing for Fishing, livestock for Livestock).

3.1.1.1 Interviews

To better understand the survey results, the findings from the non-structured interviews conducted with four community leaders and two regular community members are presented, all of whom have lived in the community for a long time. Below is a summary of the main points discussed.

Most interviewees agree that the most significant changes in the climate have been droughts, lack of rainfall, and rising temperatures, which they attribute to global warming. They also point out that climate change has negatively impacted their community, leading to a decrease in totora (a local plant), loss of fish, and droughts that reduce agricultural production, affecting crop planting and livestock feeding. Pollution has harmed aquatic wildlife, while a decline in tourism has reduced their income, limited access to food, and affected their daily lives, including health and economic activities.

All six interviewees agree that the quantity and quality of water in the lake have diminished and worsened, mainly due to contamination from garbage, sewage, and mining waste, resulting in increased algae presence.

They consider the most pressing environmental issues for their community currently to be drought, loss of fish, declining lake levels, garbage accumulation, global warming, and lack of community communication. Additionally, they have adopted traditional adaptation practices, such as seeking help from their gods for rain, preserving food during droughts, adjusting their planting schedules, and incorporating previously submerged land into new cultivation areas.

Among the most significant rituals is the one performed by the Yumani community on the Island of the Sun to invoke rain. The ceremonial procession begins with dancers adorned in traditional attire, singing chants and playing instruments as they carry a clay pot to the Peruvian side, where they must find a frog. The climax occurs on the Bolivian side of the island, at the top of the “Pallasca” viewpoint. There, the amphibian is placed inside the pot with the belief that its croaking will attract precipitation; once the water fills the container, the frog will be allowed to escape. ^45^ They hope for new rainfall to address these challenges and recognize the need to improve water quality, secure financial support, implement investment projects, conduct community clean-ups, and provide seeds and irrigation systems to strengthen their resilience against climate adversity.

In this way, their prayers in Aymara plead with God to send rain for their crops. They hope for new rains to tackle these challenges and acknowledge the need to improve water quality, receive financial support, implement investment projects, carry out community clean-ups, and provide seeds and irrigation systems to strengthen themselves against climate adversity.

Finally, the interviewees mention that they confront environmental challenges through traditional practices and prayers, without incorporating new techniques. They feel they need more financial support, better environmental education, resources such as water and productive projects, as well as greater coordination with authorities to tackle the effects of climate change. They foresee a future of continuous migration for community members (mainly men and youth) to other regions of the country or abroad due to the impacts of climate change. ^46, 47^

This is reflected in the words of community member Ismael Sillero from the Bay of Cohana, who expressed, “We live from livestock and cheese, but there’s no forage, no rain, nothing. So, one starts selling their animals. What can you do? There’s nothing left but to take another path”. ^48^

3.1.2 Participatory social mapping

The event took place in the Quehuaya community, which, due to its geographical location relative to the lake, is one of the areas most affected by decreasing water levels. The activity involved participation from all community members, including men, women, and young students from the local school.

Upon arrival, a motivational talk was given, highlighting climate change, historical data from the Binational Authority of Lake Titicaca, and a quick training session on how to create a “speaking map” organized by four groups formed for this purpose.

Before starting, the participants were shown a satellite image of the area, where they marked zones of drought, flooding, reduced agricultural, fishing, and livestock production, and affected tourist areas, noting the overall impact of climate change on their community. This collaborative work involved all participants ( Figure 3), who used different colors to indicate the areas impacted and the types of climate effects experienced. After completing the map, a secretary or spokesperson was chosen to explain the drawn map ( Figure 4).

**Figure 3.**
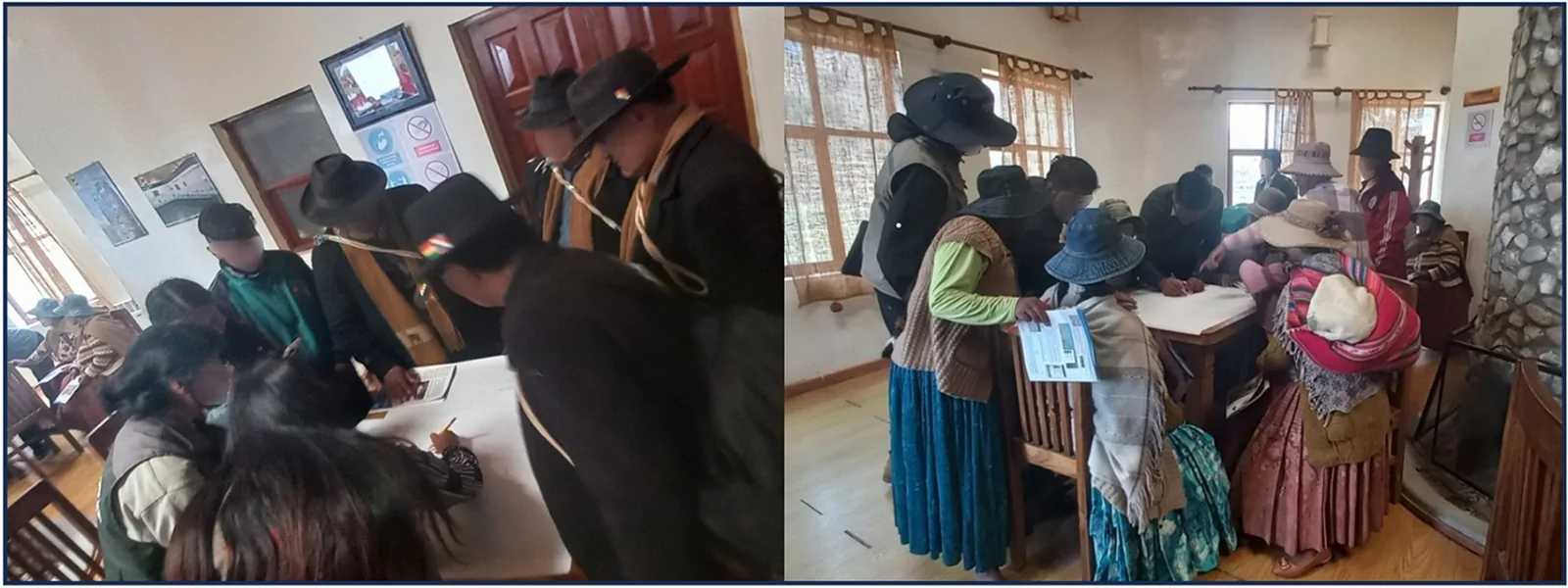
Participation of community leaders and women in the development of talking maps in the Quehuaya Community. Note: Persons shown have been de-identified.

**Figure 4.**
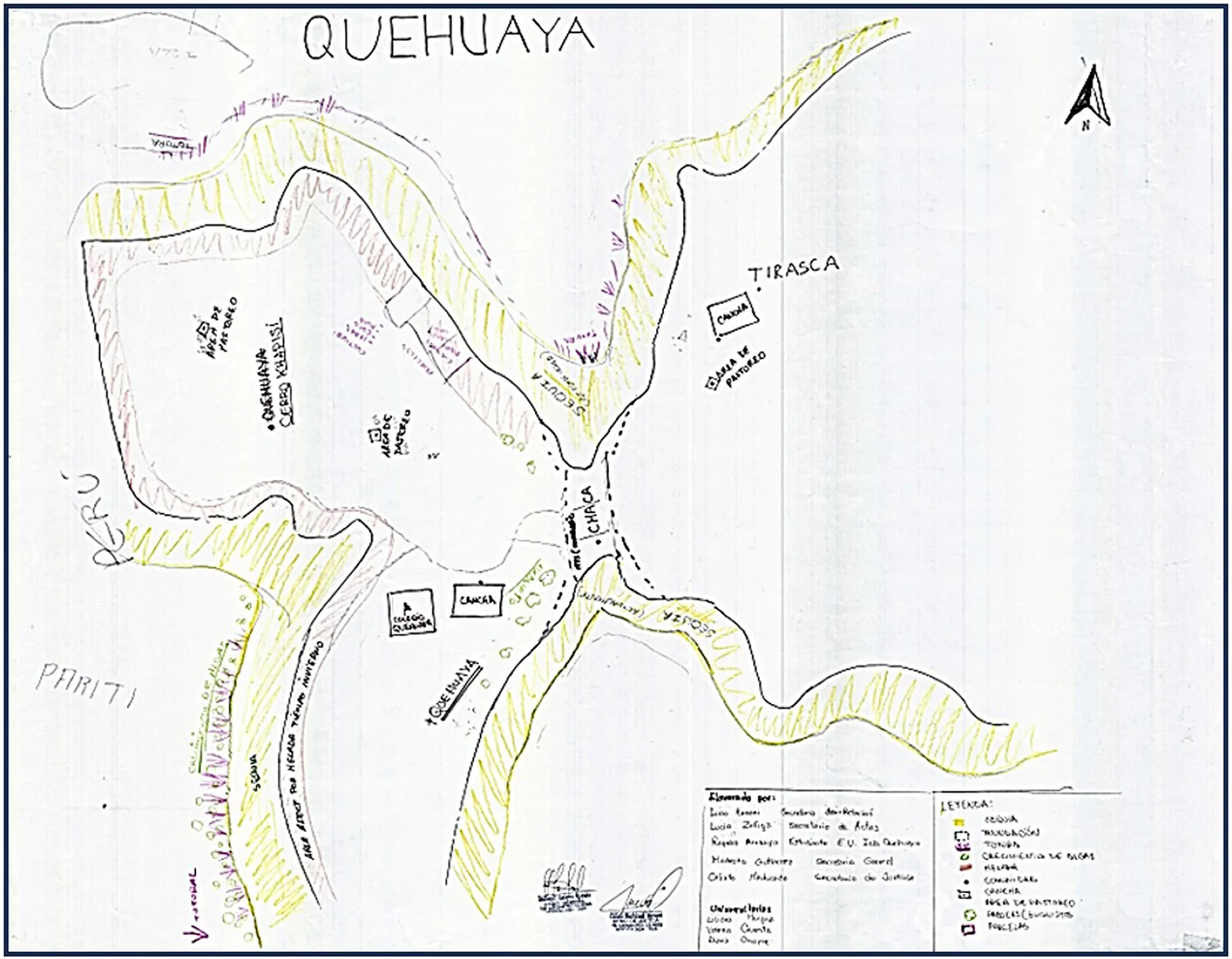
Example of a talking map created by the community leaders group.

Based on the maps created ( Figure 5), the community members shared their experiences of overcoming these adverse phenomena and the strategies they have implemented to face them. They also discussed how they have mitigated these effects or how they are adapting to these challenges.

**Figure 5.**
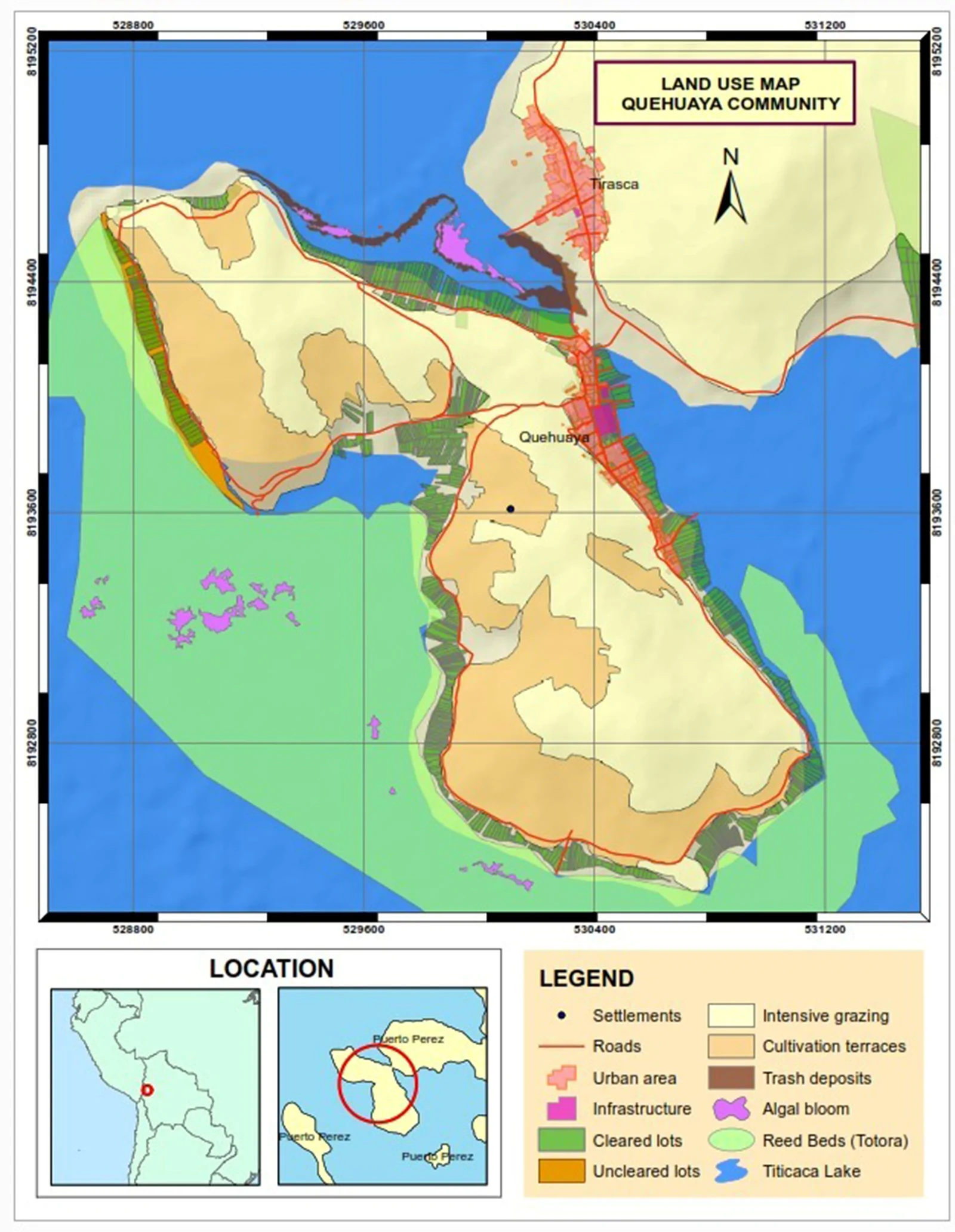
Systematization and synthesis of talking maps of the Quehuaya community.

As a result, all the information depicted in the participatory maps has been systematized and summarized into a single map ( Figure 4). This map highlights the areas of the lake’s recession, cultivated areas, and land reclaimed from Lake Titicaca for growing tubers, along with previous tourist zones and areas of decreased fishing, among others. This cartography has also been printed and provided to the community members so they can identify their future intervention spaces for new projects.

### 3.2 Workshop with promoters and agents of ecotourism and experiential tourism in Puno

As a result of conducting 26 surveys with participants at the workshop organized by MINCETUR, which included eco-tourism promoters and agents from the lakeside communities around Lake Titicaca, several key findings emerged (Appendix D).

All respondents reported experiencing significant climate changes that have impacted their tourism activities in recent years. Specifically, 92% identified a decrease in rainfall as one of the most notable changes, alongside rising temperatures and fluctuations in the lake’s water level. The sustained decline in Lake Titicaca’s water level particularly affects access and the operation of boats and floating accommodations, ultimately reducing the space available for tourism activities. Additionally, 81% noted an increase in seasonal rainfall, which involves intense downpours that damage infrastructure and alter tourist routes. Furthermore, 42% reported experiencing stronger winds and frosts, especially during winter, complicating transportation and safety for both tourists and locals.

When asked about the impact of these changes on their businesses or jobs, 96% indicated that the primary consequence is a decrease in visitors during adverse weather conditions, leading to a reduction in economic income for local communities and tourism enterprises. A similar percentage mentioned increased operational costs, necessitated by the need to reinforce vulnerable infrastructures such as boats and accommodations to withstand extreme weather conditions. A significant concern raised by 88% of respondents was the limitation of their service offerings; certain outdoor products and experiences become unfeasible under specific weather conditions, affecting both tourist satisfaction and the competitiveness of local communities. Additionally, 73% reported that safety is compromised as transportation routes and traditional itineraries become unsafe or unmanageable, forcing modifications or cancellations of scheduled activities.

In response to these challenges, 96% of participants indicated that communities and entrepreneurs have implemented various adaptation and mitigation measures. Notably, they have adjusted tourism seasons to promote activities during more favorable weather periods. Furthermore, 81% highlighted the diversification of tourism offerings, incorporating cultural, artisanal, and educational activities that are less dependent on the weather. This strategic shift allows for sustained activity under various conditions, alongside initiatives to build resilient infrastructure, such as retaining walls and boats modified for the shallower lake conditions. Finally, 77% supported the alteration of routes and itineraries to mitigate vulnerability to adverse weather phenomena.

During the seminar with MINCETUR, 73% of respondents indicated that several communities have received governmental assistance in the form of funding for resilient infrastructure projects and the modernization of tourism services, as well as training on risk management, sustainability, and community capacity building. The additional primary external support sources for tourism development included NGOs (35%), universities (27%), and the private sector (15%).

When asked about additional support deemed essential for strengthening tourism resilience in their region, 100% of participants emphasized the need for greater financial investment in resilient infrastructure, including protective walls, suitable boats, and improvements to sanitation systems. Furthermore, 88% called for enhanced coordination between public and private institutions and communities to enable more integrated and effective responses, alongside the provision of adequate, systematic, and updated technical assistance. Meanwhile, 73% emphasized the significance of access to real-time climate information and accurate forecasts for effective planning and informed decision-making. Regarding barriers to accessing these supports, 23% highlighted the need for streamlining permits and regulations to facilitate the swift implementation of improvements without encountering excessive bureaucratic obstacles.

## 4. Discussion

The results confirm that climate change is manifesting in the surrounding area of Lake Titicaca as a socio-ecological syndrome with multiple stressors: a sustained decline in lake levels, ^49, 50^ increased intra-annual rainfall variability, rising temperatures, ^51^ intensified winds, and extreme events. In line with recent literature, these changes operate cumulatively on fragile ecosystems and water-sensitive livelihoods, amplifying historical vulnerabilities and pre-existing inequalities. ^9, 17, 35^

Firstly, the generalized perception of water level loss of Titicaca (100%), along with contamination, confirms a structural rather than a temporary water crisis. The perceived drought and the community prioritization of irrigation and storage highlight a gap between water supply and productive demand, leading to declining yields and the shifting of crops to newly exposed soils.

Secondly, tourism findings confirm a “domino effect” between the declining lake level, operational restrictions (access, navigability), and falling demand, resulting in increased costs and risks. Diversifying offerings and adjusting seasons are emerging as early adaptive responses, but they remain reactive and limited in scope unless accompanied by resilient infrastructure, accessible insurance, and timely and useful alert systems.

Thirdly, participatory social mapping reveals an accelerated reconfiguration of the territory: retreating water surfaces, expansion of crops on newly exposed soils, and contraction of riverine habitats. This “advance on the banks” demonstrates short-term resilience but may compromise ecological functions (wetlands as buffers, water quality) and increase future exposure to floods or socio-environmental conflicts. ^52^

In summary, the evidence indicates that the system has entered a state of chronic water deficit, necessitating a shift from reactive responses to integrated management: technical irrigation and decentralized storage, restoration of regulatory wetlands, ^53^ pollution control, ^54^ and binational agreements for allocation and early warning to sustain water and productive security.

## 5. Conclusions

Research on the impacts of climate change on communities around Lake Titicaca has revealed and confirmed a complex and concerning reality. The findings indicate that climate change is causing a chronic water deficit in the region, as evidenced by declining lake levels, increasing droughts, and climate variability affecting various productive activities, including agriculture and fishing. This situation not only threatens the food and economic security of the Aymara communities and other lakeside groups, but also interferes with public health and exacerbates existing socio-economic vulnerabilities.

Through a participatory approach, there is a high level of community awareness regarding climate change and a willingness to adapt to the new reality through traditional practices. However, the responses observed are reactive and limited, highlighting the urgent need to implement more integrated and sustainable adaptation policies. The importance of promoting efficient water management, restoring critical ecosystems like wetlands, and establishing transboundary coordination mechanisms to ensure water security is emphasized.

Adaptation strategies must be accompanied by significant resources, continuous training, and greater integration between communities, governments, and organizations. The creation of resilient infrastructure and early warning systems is essential to mitigate the effects of climate change and enhance the resilience of the communities surrounding Lake Titicaca.

This study integrated a social component based on knowledge exchange workshops and participatory mapping. It has been compared with climatological and aerospace information, highlighting the gap between Western knowledge and ancestral practices rooted in direct observation of nature. These traditional practices involve strong community participation led by the Pachayatiri, a local expert responsible for forecasting the weather and preventing risks (such as droughts or frosts) using bioclimatic, astronomical, and atmospheric indicators. ^45, 55^ However, the project revealed that these indicators are being altered due to climate change, as the occurrence of extreme and irregular events disrupts natural signals, making it more difficult to predict their effects through traditional knowledge and practices. ^56^

In conclusion, the findings of this research stress that Lake Titicaca, as an integral socio-ecological system, requires a holistic approach that considers not only adaptation to environmental changes but also the promotion of equitable and sustainable development that preserves its cultural and ecological value.

## Data Availability

The following information presents the primary and intermediate processed data that support the results of this article. These data are hosted in the Zenodo repository, which can be freely accessed at Primary data from an article in the journal 1000Research [Data set]. https://doi.org/10.5281/zenodo.18521682. ^57^ Data are available under the terms of the Creative Commons Attribution 4.0 International license (CC-BY 4.0).
