# The perception of the impacts of climate change in communities surrounding Lake Titicaca, Peru-Bolivia.

**DOI:** 10.12688/f1000research.176641.2

**Published:** 2026-05-26

**Authors:** Alberto Enrique Garcia Rivero, Yuri Vladimir Sandoval Montes, Edwin Natividad Gabriel Campos, Javier Alfredo Núñez Villalba, Zaniel Israel Novoa Goicochea

**Affiliations:** 1 Universidad Nacional Mayor de San Marcos Facultad de Ciencias Sociales, Lima District, Lima Region, Peru; 2 Universidad Mayor de San Andres Facultad de Ingenieria, La Paz, Bolivia; 3 Pontificia Universidad Catóica del Perú Departamento de Humanidades, San Miguel, Lima, Peru

**Keywords:** climate change; drought; precipitation; fishery; agriculture; adaptation, mitigation; Lake Titicaca.

## Abstract

**Background:**

Climate change is increasingly transforming socio-ecological systems in high-Andean regions, particularly in communities surrounding Lake Titicaca, where livelihoods depend heavily on climate-sensitive activities such as agriculture, fisheries, livestock, and tourism. Despite growing environmental pressures, limited evidence exists regarding how local populations and institutional actors perceive climate-related impacts and adaptive responses in this transboundary region. This study analyzes climate change perceptions, perceived impacts, and adaptation capacities in communities located in Puno Bay (Peru) and Cohana Bay (Bolivia).

**Methods:**

A mixed-methods approach integrating quantitative and qualitative techniques was applied during fieldwork conducted in November 2024 and June 2025 in the communities of Quehuaya, Capachica, Guaqui, and Puno. Data collection included structured surveys administered to 62 community members and 26 institutional actors, semi-structured interviews, participatory workshops, and participatory mapping exercises. Descriptive statistics, chi-square tests, and Spearman correlations were used for exploratory quantitative analysis, while qualitative information was analyzed through thematic coding and socio-ecological interpretation.

**Results:**

Findings revealed high levels of climate change awareness among community members (85.5%) and institutional actors (100%). Respondents identified reduced rainfall, increasing temperatures, hydrological variability, declining agricultural productivity, reduced fish availability, and tourism-related impacts as the principal perceived effects. Significant differences were identified regarding adaptation measures (χ ^2^ = 14.96, p < 0.001) and institutional support (χ ^2^ = 18.32, p < 0.001), evidencing structural inequalities in adaptive capacity. A moderate positive correlation (ρ = 0.36, p = 0.003) was observed between climate perception and reported impacts.

**Conclusions:**

Climate change is widely recognized across Lake Titicaca communities; however, adaptive capacity remains uneven due to disparities in institutional support, resources, and technical knowledge. Strengthening multi-level governance, participatory adaptation strategies, and transboundary cooperation is essential for improving resilience in Andean socio-ecological systems.

**Abbreviations**
ALT: Binational Authority of Lake Titicaca
CONCYTEC: National Council of Science, Technology and Innovation of Peru
IIGEO-UMSA
: Institute of Geographic Research
MINCETUR: Ministry of Foreign Trade and Tourism of Peru
PROCIENCIA: National Program for Scientific Research and Advanced Studies

## 1. Introduction

Climate change has emerged as one of the principal drivers transforming ecosystems and altering socio-environmental systems, affecting both natural environments and human communities. Recent studies (2020–2024) have documented a wide range of impacts, including ecological degradation and increasing socio-environmental vulnerability associated with ecosystem alteration and the decline of ecosystem services. ^1^ ^–^ ^5^ In socially exposed contexts, climate risk exacerbates economic and social inequalities, thereby increasing the vulnerability of populations dependent on natural resources. ^6–9^

Climate variability, which affects precipitation patterns, temperature regimes, and hydrological balances, constitutes a critical dimension of climate change, requiring predictive models and adaptation strategies grounded in hydrometeorological evidence. ^10^ ^–^ ^13^ Likewise, climate change contributes to biodiversity alterations characterized by shifts in species distribution and abundance, accompanied by losses in ecological functionality. ^14^ ^–^ ^16^

Recent research emphasizes that climate change impacts cannot be understood solely through biophysical measurements; rather, they require integrated frameworks linking climatic drivers, environmental processes, and human responses. Socio-ecological systems theory provides a valuable perspective for understanding these interactions by highlighting the feedback mechanisms between environmental change and social adaptation processes. Within this framework, vulnerability is determined not only by exposure to climatic stressors, but also by adaptive capacity, institutional context, and access to resources.

A review of studies conducted over the last five years confirms that climate change effects manifest across multiple dimensions, including climate variability, ecological degradation, habitat loss, and increasing socio-environmental vulnerability. ^17^ ^–^ ^30^ These impacts extend beyond ecological concerns, affecting social and territorial structures and demanding integrated approaches to environmental management and territorial planning.

Lake Titicaca, the center of a vast ecological, economic, and cultural network in the Altiplano region, has experienced a marked and alarming decline in water levels over recent years. This phenomenon, driven by a combination of global and regional climatic factors together with local resource management practices, represents a structural threat to lakeside communities that depend on the lake for water supply, food security, income generation, and cultural identity. This research, funded by the PROCIENCIA program of CONCYTEC (Peru) in 2024, aims to identify and characterize the principal impacts of climate change on populations inhabiting the Lake Titicaca basin, with particular emphasis on Puno Bay (Peru) and Cohana Bay (Bolivia), while also contributing elements to guide adaptation policies and integrated transboundary management strategies.

Aymara communities and other lakeside populations maintain a close multisectoral relationship with the lake. Artisanal fisheries and small-scale agriculture constitute the foundation of food security and local livelihoods, while lake water supports human consumption, irrigation, and productive activities. In addition, landscapes and cultural practices associated with the lake sustain traditional knowledge systems, rituals, and forms of community organization. ^31^ ^–^ ^34^

The sustained reduction in water volume and increasing hydrological variability—particularly recurrent drought episodes—alter local hydrological regimes, reduce the productivity of agricultural and fisheries systems, and threaten the provision of environmental and tourism-related services. ^31^ ^–^ ^33^

This study explicitly focuses on local perceptions of climate change rather than attempting to establish direct causal relationships between climatic variables and observed impacts. Understanding perception is essential because it shapes how individuals and communities interpret environmental change, ^35^ ^–^ ^36^ prioritize risks, and implement adaptation strategies. By comparing community-level perceptions with those of institutional actors, this research identifies gaps in response capacity and governance that may affect the effectiveness of climate change responses in the Lake Titicaca region.

The multifaceted effects identified at the community level include: reduced agricultural production and crop diversity resulting from decreased water availability for irrigation; loss of biomass and fisheries habitats affecting fish catch and quality; degradation of wetlands and riparian zones that diminishes hydrological regulation capacity and biodiversity; ^34^ ^,^ ^37^ and increasing concentrations of pollutants due to lower water volumes, with significant implications for public health. ^38^ These productive and health-related impacts are accompanied by profound sociocultural consequences, including the disruption of traditional collective water management practices, erosion of knowledge associated with fishing and agriculture, and alteration of rituals linked to the lake cycle. Finally, the deterioration of livelihoods drives processes of internal and transboundary migration that reconfigure demographic, economic, and family dynamics, generating additional vulnerabilities for communities remaining in the territory.

## 2. Materials and methods

To comprehensively address perceptions of climate change impacts among lakeside communities surrounding Lake Titicaca, this research adopted a multidisciplinary and participatory approach integrating both qualitative and quantitative techniques. The methodology was designed in accordance with the objectives of identifying the perceptions of inhabitants of circumlacustrine communities regarding the principal impacts of climate change in selected communities of Cohana Bay (Bolivia) and Puno Bay (Peru), together with other areas of interest (see Figure 1), as well as providing elements capable of informing adaptation and mitigation policies in response to climate change impacts.

**Figure 1.**
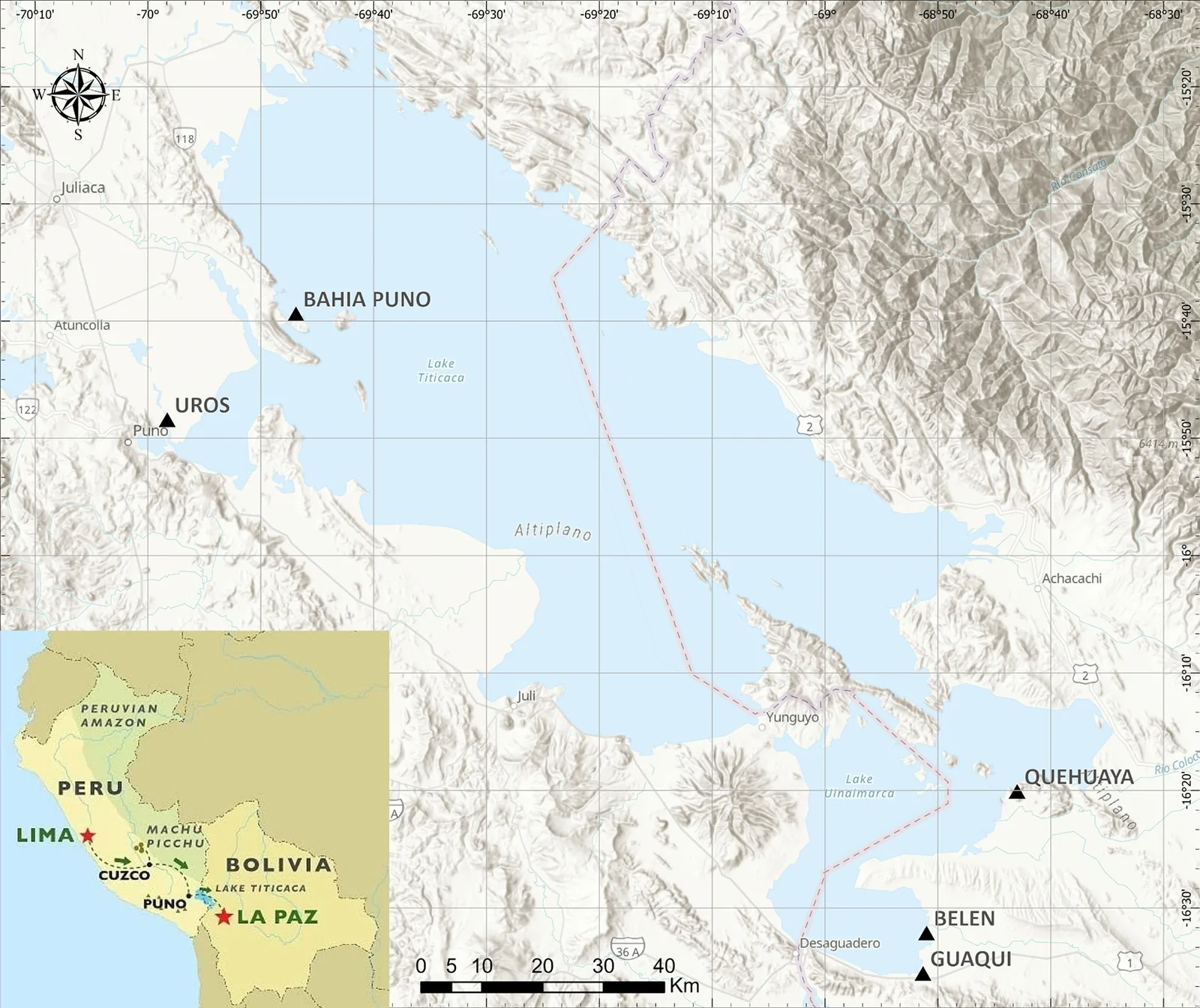
Location of the study areas for this research.

The investigation began with an exhaustive review of scientific literature, institutional reports, and historical studies related to the physical, socioeconomic, and cultural environment of Lake Titicaca and its communities, together with an analysis of climate change, climate variability, and their principal impacts. This review made it possible to contextualize observed changes within the lacustrine system, understand long-term trends, and identify factors contributing to modifications in hydrological and social regimes.

Fieldwork was conducted during two periods: November 2024 (at the end of the dry season) and June 2025 (at the beginning of the dry season). Research activities were carried out inthe communities of Quehuaya, located in Cohana Bay, and Capachica, located in Puno Bay, selected due to their representativeness and vulnerability to changes affecting the lake. Additionally, work was conducted in the Bolivian sector in Guaqui, a locality and municipality in Ingavi Province, Department of La Paz, situated on the shores of Lake Titicaca. In the Peruvian sector, the study focused on an area of the city of Puno where the principal sewage discharges flow into the lake.

This study adopts a mixed-methods approach that integrates quantitative and qualitative data to capture the complexity of socio-ecological dynamics. The quantitative component was based on structured surveys administered to community members (see Appendix A) and participants in the Ecotourism Workshop held in Puno (see Appendix B), using binary variables (presence/absence), which enabled the construction of comparable indicators across respondents. The qualitative component included semi-structured interviews with community leaders, farmers, fishers, and local authorities aimed at identifying the most relevant threats and community responses to climatic alterations and their consequences for ecosystems and local populations.

Within the framework of participatory workshops conducted in Quehuaya and Capachica, participatory mapping exercises were carried out to provide contextual insights into how environmental changes are perceived and experienced, including drought, flooding, reductions in agricultural, fisheries, and livestock production, as well as impacts on tourism areas, thereby identifying the general effects of climate change on local communities. ^39^ ^–^ ^42^ This collaborative work involved all participants ( Figure 2), who used different colors to identify affected areas and the types of climatic effects experienced. Upon completion of the maps, a secretary or spokesperson was selected to explain the resulting map.

**Figure 2.**
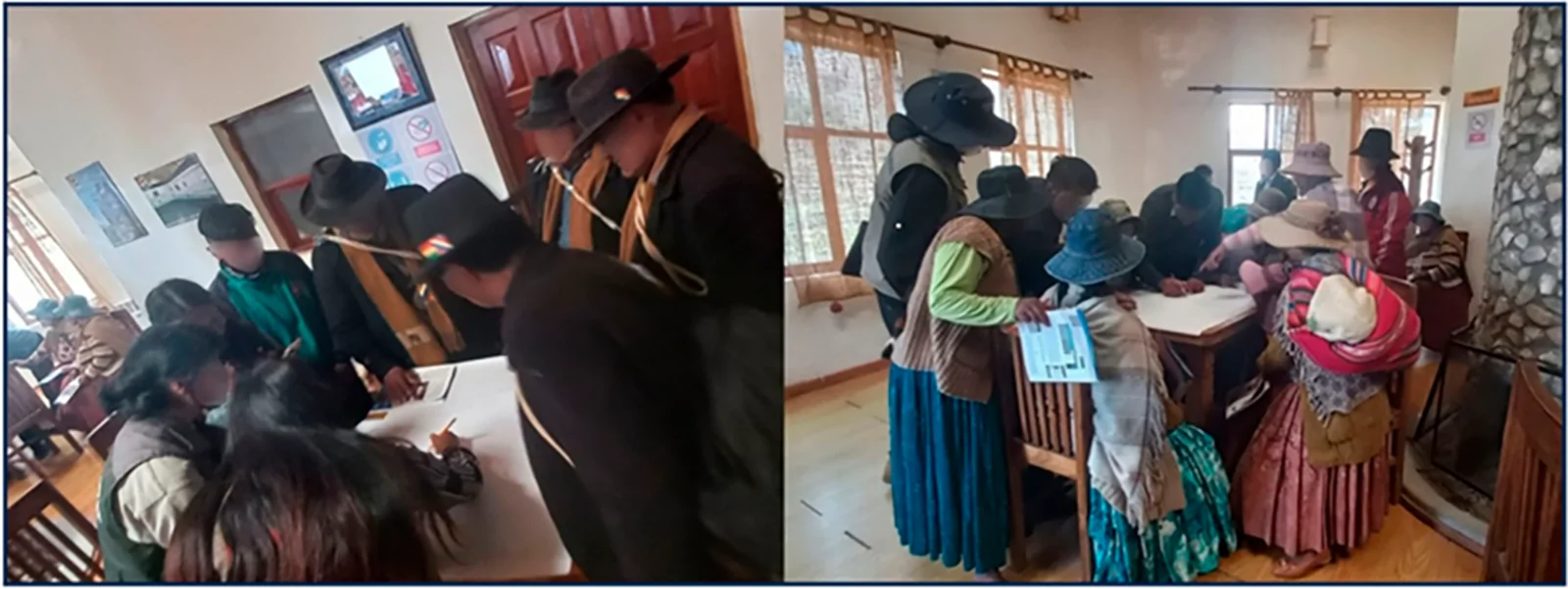
Comparative multi-actor analysis of climate change perception, impacts, adaptation, and institutional support.

The sampling strategy was purposive and non-probabilistic, designed to capture variability across communities with different levels of exposure and dependence on climate-sensitive livelihoods. Selection criteria included geographic representation, economic activities, and accessibility. The final sample consisted of 62 community respondents from the Quehuaya and Capachica Communities and 26 institutional actors associated with tourism and environmental management who participated in a Workshop on Ecotourism in the City of Puno organized by MINCETUR. Although this sampling approach limits statistical representativeness, it is appropriate for exploratory socio-ecological analysis and hypothesis generation.

Data analysis combined descriptive statistics with exploratory inferential techniques. Chi-square tests were used to assess differences between groups, while Spearman correlations were applied to examine relationships between perception and reported impacts. Qualitative data were analyzed through thematic coding, identifying key categories such as water stress, livelihood impacts, and adaptation strategies. All statistical analyses were conducted as exploratory because of the sample size and the binary structure of the dataset. ^43^

Composite indices were constructed by aggregating binary variables within thematic groups, allowing for a standardized representation of socio-ecological responses. The defined ranges reflect the number of variables included in each index and enable comparison across respondents and actor groups within an exploratory analytical framework ( Table 1).

**Table 1.** Composite indices used to summarize perception, impacts, adaptation, and support dimensions.

Index	Description	Range
Climate perception index	Number of perceived signals	0–5
Impact index	Reported impacts	0–5
Adaptation index	Adaptation measures	0–4
Support index	Institutional support sources	0–4
Needs index	Identified needs	0–6

It should be emphasized that, because the activities included participatory workshops with community members aimed at collecting traditional knowledge, perceptions regarding changes in the lake, and existing adaptation strategies, all activities were conducted in strict compliance with the “Code of Ethics in Research of the Universidad Nacional Mayor de San Marcos,” approved through Rectoral Resolution No. 012648–2023-R/UNMSM of November 21, 2023.

For this study, verbal informed consent was obtained from the communities where the research was conducted. In these communities, consent is traditionally granted verbally; accordingly, consent was obtained following the presentation, explanation, and agreement with community leaders regarding the project objectives, principal activities, and expected benefits prior to the initiation of the study through a preliminary visit to the research area. Tacit and practical confirmation of such consent was evidenced through the participation of community members and leaders in the various workshops and field visits conducted within the study area. In summary, the sole valid reason for employing verbal informed consent was that the communities involved in this study do not customarily provide written and signed consent, as oral communication and personal commitment carry greater social significance within these populations.

## 3. Results

The responses from the 62 surveys administered to community members during the community workshops in Quehuaya and Capachica are summarized in Appendix C–Table C1.1 and Table C2.1, categorized by gender and productive activity, respectively. The sample consisted of 33 women and 29 men, all over 16 years of age, with an average age of 48 years. Regarding educational level, 66% of respondents had not completed primary education, 24% had completed secondary education, and only 13% had undertaken technical or university studies.

Similarly, the responses from the 26 surveys administered during the Ecotourism Workshop held in the city of Puno and organized by MINCETUR are summarized in Appendix D.

### 3.1. Climate change perception

The results indicate consistently high levels of climate change perception among both community members and institutional actors. Respondents frequently identified reduced rainfall, increased temperature, and hydrological variability as key indicators of environmental change. These perceptions reflect shared experiences of environmental change across actor groups. ( Table 2).

**Table 2.** Comparative indicators of climate change perception, impacts, adaptation, and institutional support.

Dimension	Communities (%)	MINCETUR (%)	Difference
Climate change perception	85.5	100.0	+14.5
Impact recognition	95.2	96.2	+1.0
Adaptation measures	53.2	96.2	+43.0
Institutional support	33.9	84.6	+50.7
Support needs	93.5	100.0	+6.5

Table 2 reveals key differences between community respondents and institutional actors, point to a governance gap in adaptive capacity. ^35^ ^,^ ^44^

Despite differences in knowledge systems and access to information, both groups converge in their recognition of climate-related changes. This convergence suggests that climate change has become a salient issue in the region, shaping both local experiences and institutional narratives. However, although perception levels are similar, the implications of these perceptions differ substantially in terms of response capacity.

Figure 3 shows that, although perception and impact recognition are similarly high across groups, adaptation and institutional support differ markedly. This contrast reveals structural inequalities in access to resources, information, and governance mechanisms.

**Figure 3.**
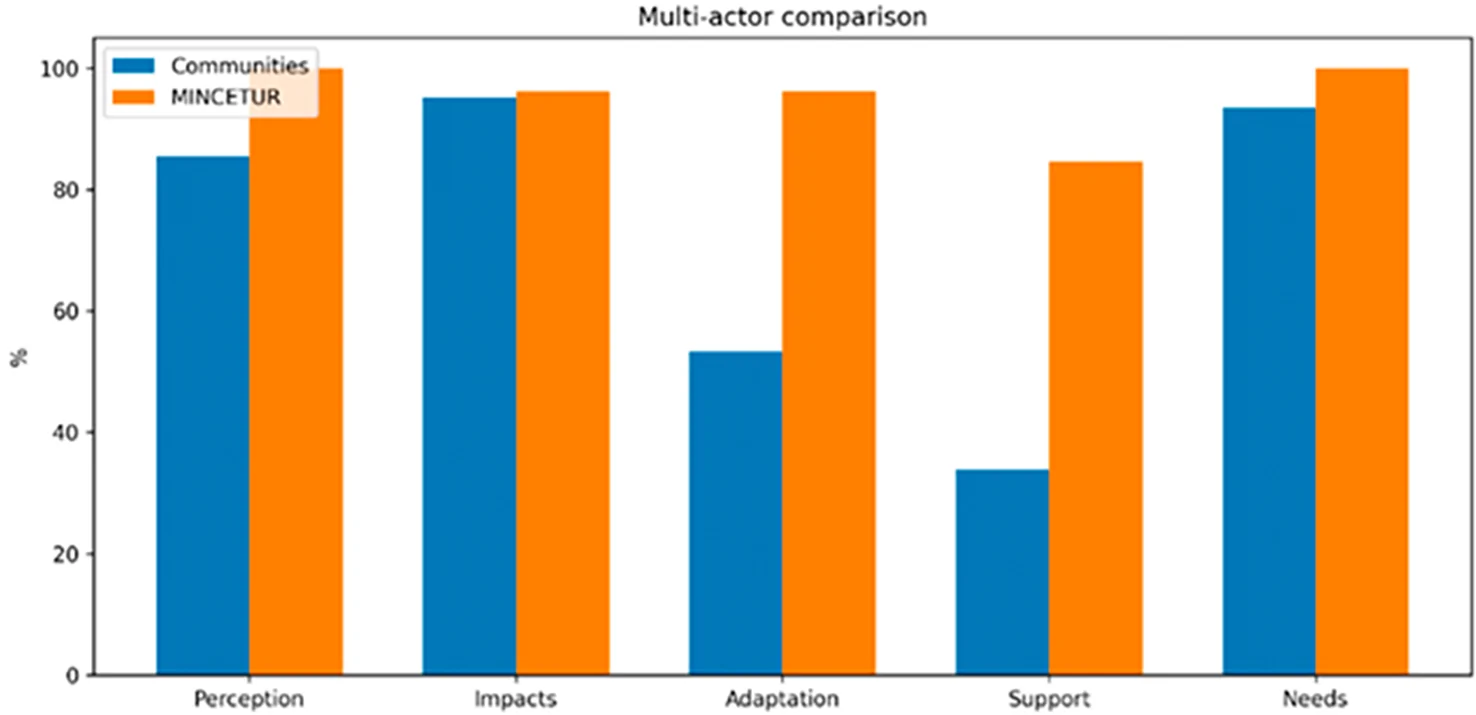
Participation of community leaders and women in the development of talking maps in the Quehuaya Community. Note: Persons shown have been de-identified.

The results presented in Table 3 provide exploratory statistical evidence supporting the differences observed between community and institutional actors. ^45^ The chi-square tests indicate statistically significant differences in both adaptation (χ ^2^ = 14.96, p < 0.001) and access to institutional support (χ ^2^ = 18.32, p < 0.001), confirming that these dimensions vary systematically between groups rather than occurring by chance. The stronger chi-square value for institutional support suggests a particularly pronounced disparity in access to resources and support mechanisms.

**Table 3.** Statistical analysis.

Test	Statistic	p-value	Interpretation
Chi-square (adaptation vs group)	χ ^2^=14.96	<0.001	Significant
Chi-square (support vs group)	χ ^2^=18.32	<0.001	Strong disparity
Spearman (perception-impact)	ρ=0.36	0.003	Moderate correlation

In addition, the Spearman correlation (ρ = 0.36, p = 0.003) indicates a moderate positive association between climate change perception and reported impacts. This suggests that higher levels of awareness are linked to greater recognition of environmental and socio-economic effects. However, this relationship does not necessarily translate into greater capacity to adapt, reinforcing the interpretation that adaptation is influenced by structural and institutional factors beyond perception alone.

Overall, these results support the central argument of the study by providing quantitative evidence of a governance gap: while perception and impact recognition are relatively aligned across groups, adaptation and institutional support remain unevenly distributed. Because these analyses are exploratory, they should be interpreted as indicative patterns rather than definitive causal relationships.

### 3.2. Perceived impacts and adaptation

Perceived impacts are widespread across multiple livelihood sectors, including agriculture, fisheries, livestock, and tourism. Respondents reported reduced agricultural productivity, changes in seasonal cycles, declining fish availability, and disruptions to tourism activities. These findings indicate that climate variability is understood not only as an environmental issue, but also as a direct driver of socio-economic stress.

In contrast to the high level of perceived impacts, adaptation responses show significant variability. Approximately half of community respondents reported implementing adaptation measures, indicating moderate adaptive capacity. ^35^ ^,^ ^44^ Institutional actors reported substantially higher levels of adaptation, reflecting differences in access to knowledge, resources, and organizational support. This disparity reveals a structural imbalance in the ability to respond to environmental change.

Figure 4 shows the frequency of reported climatic indicators, including reduced rainfall, increased temperature, and hydrological variability. The high prevalence of these signals reflects strong consensus among institutional actors and indicate the role of technical knowledge and information access in shaping climate awareness. ^35^ ^–^ ^36^

**Figure 4.**
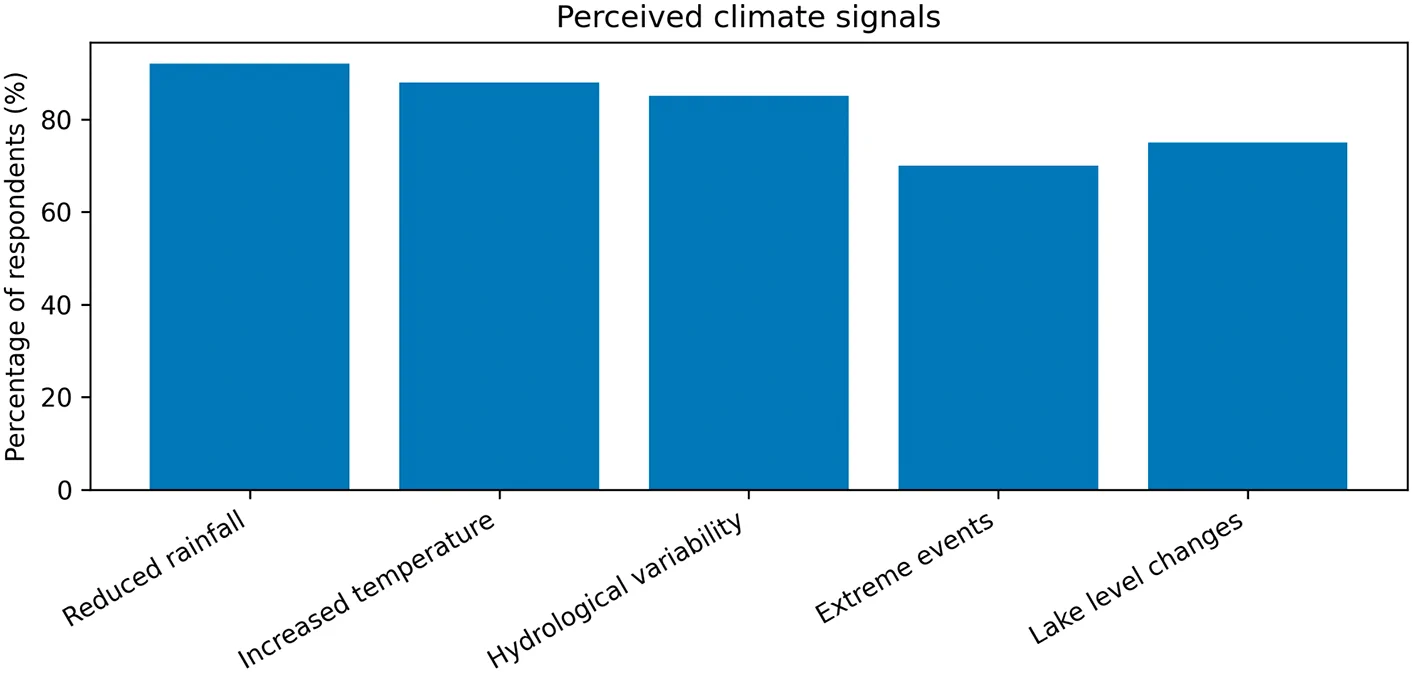
Perceived climate change signals reported by institutional actors.

Figure 5 illustrates the distribution of reported impacts across key sectors. Climate variability is perceived as a direct socio-economic stressor that affects income stability and reinforces the linkage between environmental change and livelihood vulnerability.

**Figure 5.**
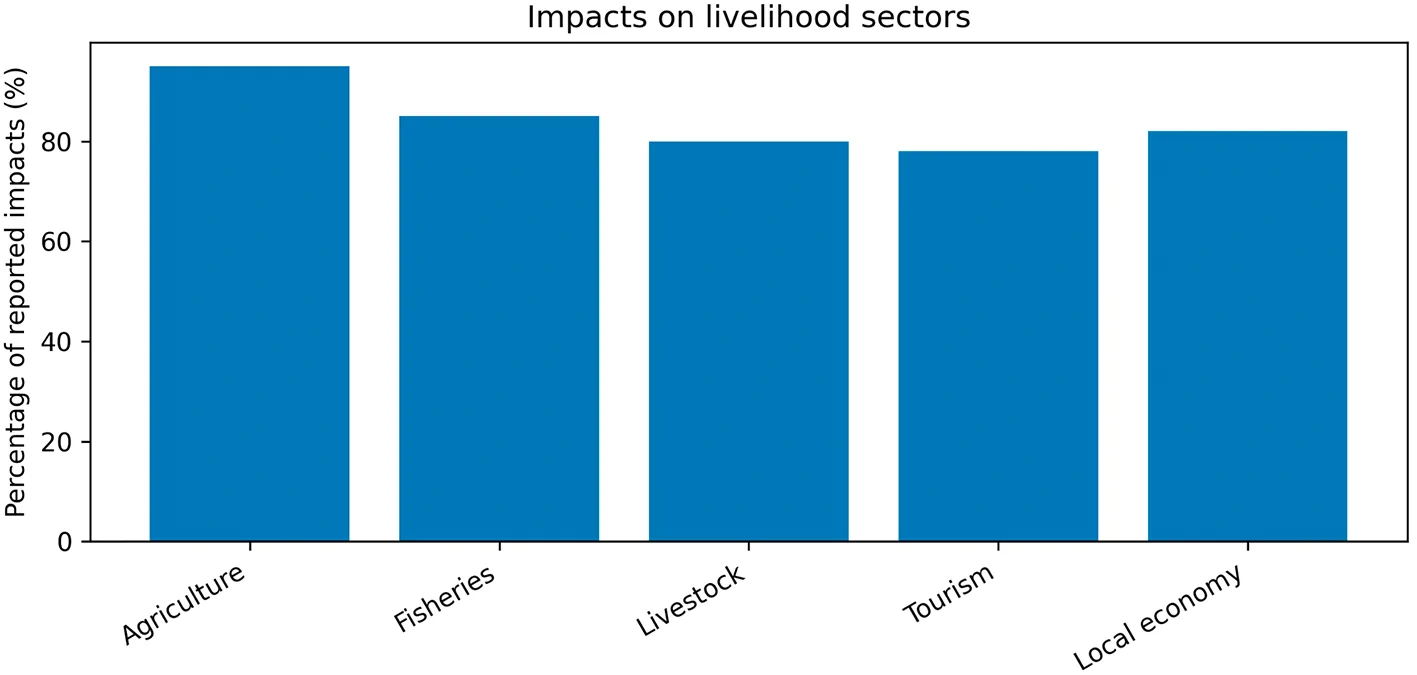
Perceived impacts of climate change on economic and livelihood sectors.

Figure 6 presents the reported adaptation strategies implemented by respondents. The distribution of responses suggests that adaptation potential is strongly influenced by access to knowledge, resources, and institutional support.

**Figure 6.**
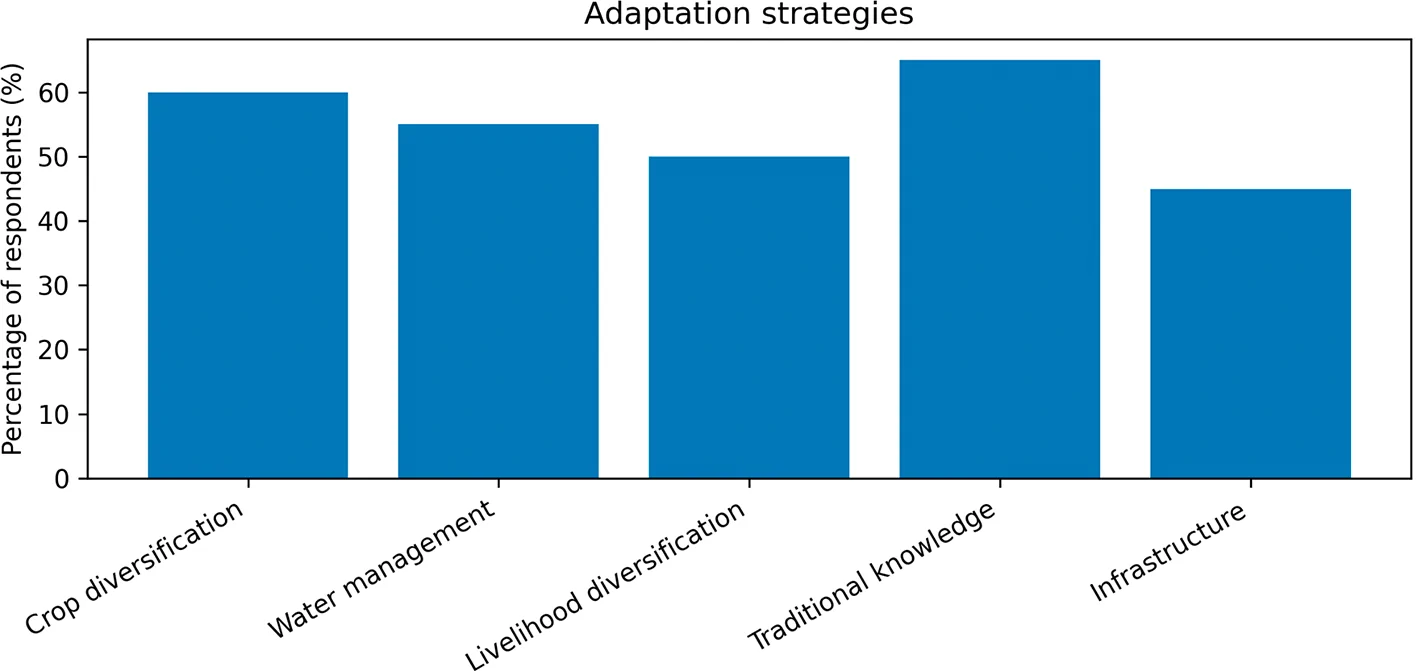
Reported adaptation strategies among respondents.

Institutional support was primarily associated with the sources of institutional support reported by participants. The predominance of governmental and organizational actors indicates that adaptation processes are mediated by formal support structures, although variability in support sources suggests fragmentation that may limit effectiveness ( Figure 7).

**Figure 7.**
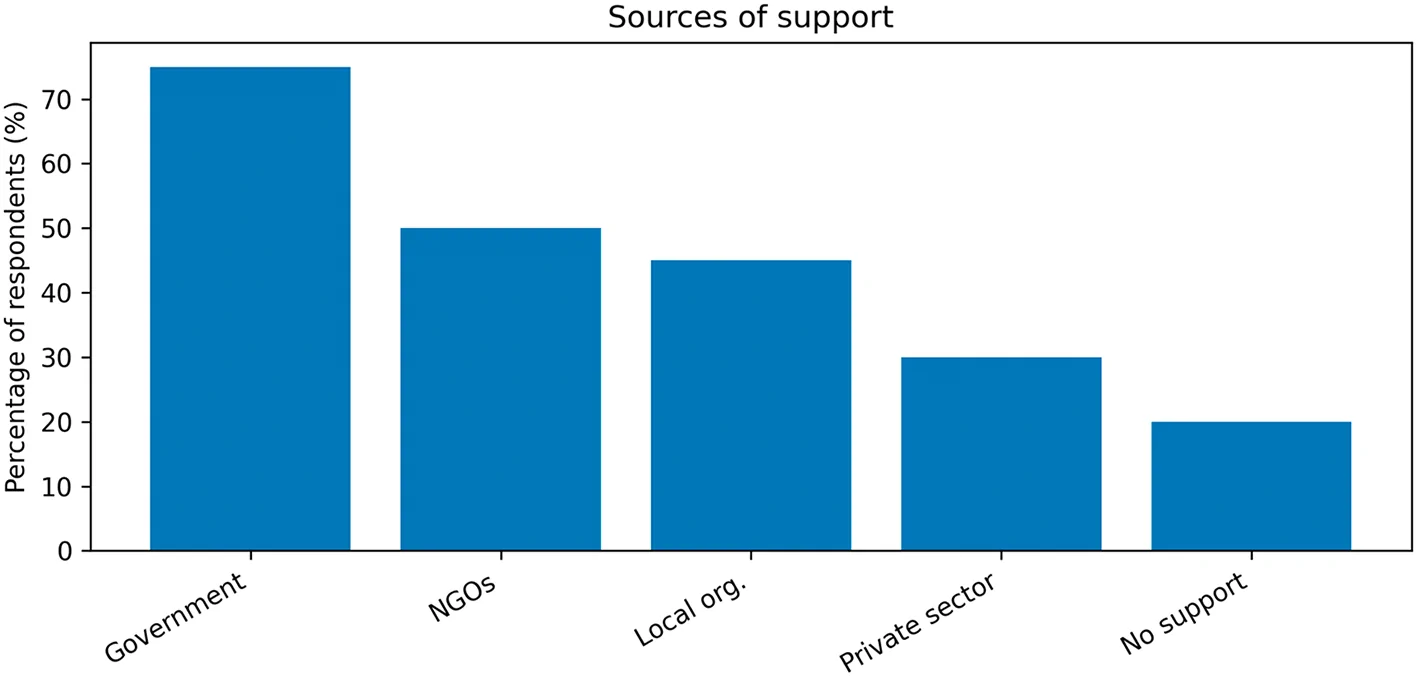
Sources of institutional support reported by participants.

Figure 8 presents the identified needs for strengthening capacity to respond to climate change. The diversity of needs expressed by respondents indicates a clear mismatch between perceived risks and available resources, emphasizing the need for integrated and multi-level adaptation strategies.

**Figure 8.**
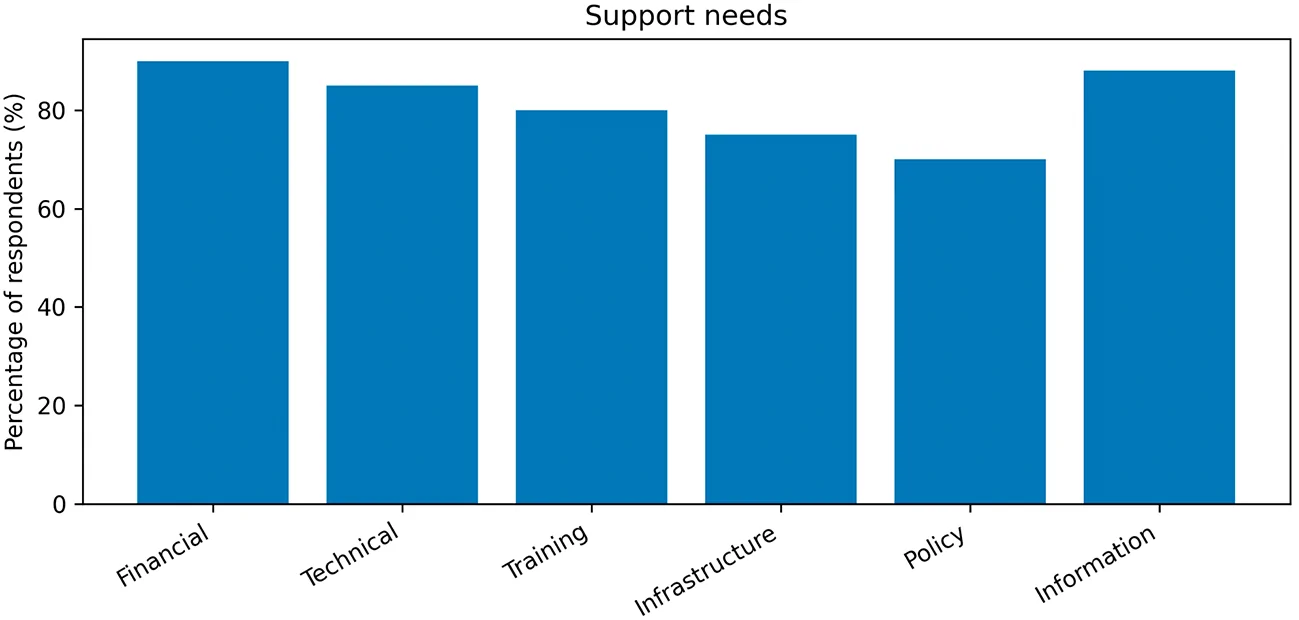
Identified needs for strengthening climate change response capacity.

Figure 9 point to the diversity of support needs expressed by respondents. These results point to a mismatch between perceived climate-related risks and available adaptive resources, reinforcing the need for coordinated financial, technical, and institutional interventions.

**Figure 9.**
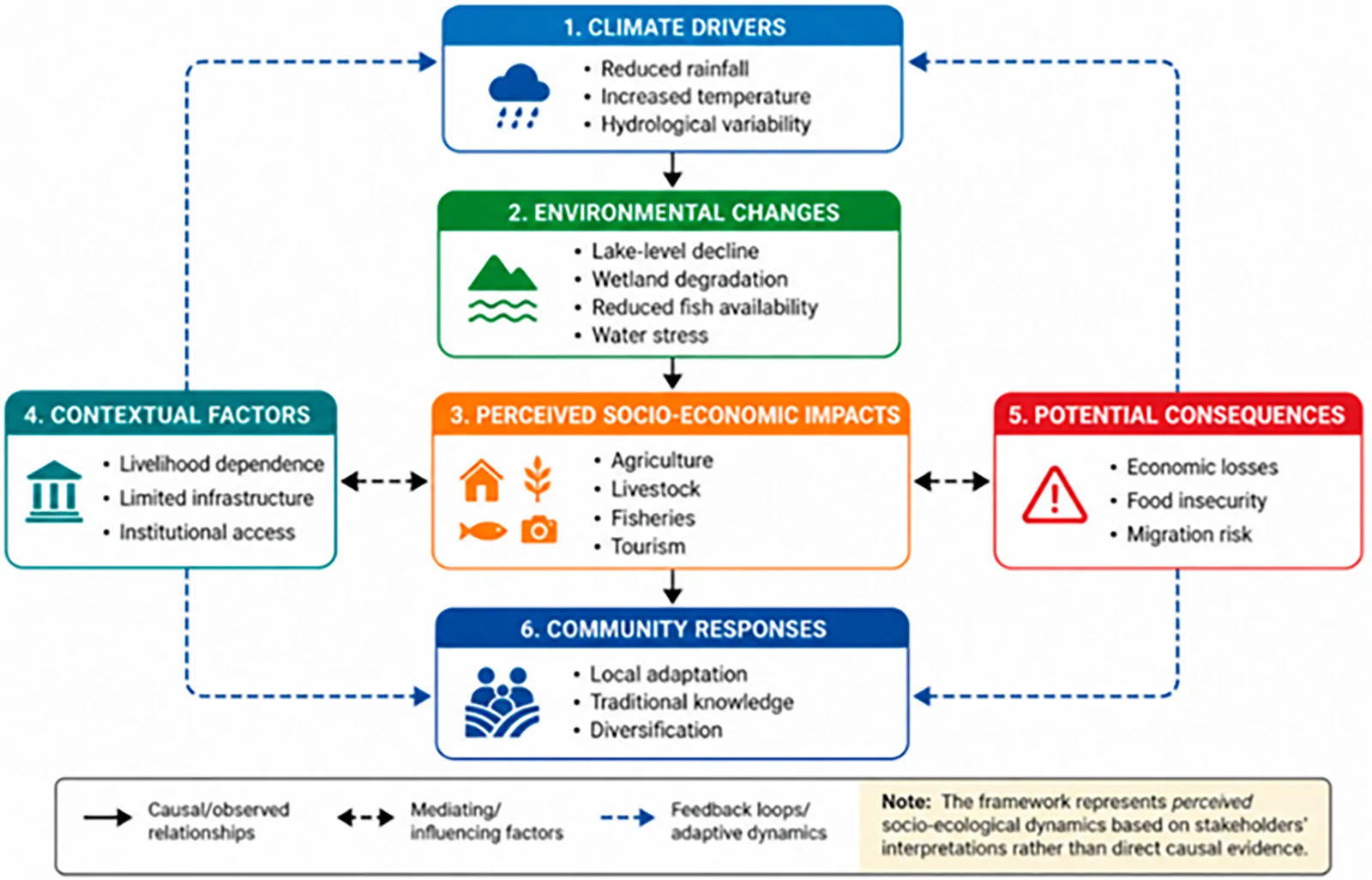
Socio-ecological conceptual framework linking climate drivers, environmental change, and adaptive responses in Lake Titicaca communities.

The results summarized in Table 4 provide a synthetic overview of the responses reported by institutional actors across key thematic dimensions. High levels of climate change perception and impact recognition confirm a consolidated understanding of environmental change within this group. Similarly, the high level of reported adaptation measures suggests a relatively strong adaptive capacity among institutional stakeholders.

**Table 4.** Responses reported by institutional actors across key thematic dimensions.

Block	Level	Max	Interpretation
Climate signals	High	5	Strong perception
Impacts	High	5	Widespread recognition
Adaptation	High	4	High capacity
Support	Moderate	4	Uneven support
Needs	Very high	6	Strong demand

This table synthesizes the distribution of responses across key thematic dimensions among institutional actors, highlighting strong alignment between climate change perception, impact recognition, and reported adaptation capacity. However, the moderate level of institutional support, combined with very high levels of identified needs, reveals a structural imbalance, indicating that even within institutional contexts, access to resources and effective support mechanisms remains uneven.

At the same time, the very high level of identified needs suggest a persistent gap between perceived risks and available support. This pattern reinforces the interpretation that, despite high awareness and response capacity, structural constraints continue to limit the effectiveness of climate adaptation processes.

Complements the quantitative findings by providing a qualitative synthesis of key themes identified through interviews and participatory methods. The results underscore the central role of water stress as a perceived environmental challenge, together with its direct implications for livelihood systems such as agriculture, fisheries, and tourism ( Table 5).

**Table 5.** Qualitative synthesis.

Category	Findings
Water stress	Perceived reduction in water availability
Livelihood impacts	Agriculture, fisheries, tourism affected
Adaptation practices	Traditional and incremental strategies
Environmental change	Shoreline retreat and ecosystem shifts

Table 5 provides a qualitative synthesis of key socio-ecological themes emerging from interviews and participatory methods, emphasizing the central role of water stress and its cascading effects on livelihood systems. The findings reveal that adaptation strategies are predominantly incremental and rooted in local knowledge, reflecting both the adaptive agency of communities and the limitations imposed by restricted access to institutional support and resources.

In addition, the findings emphasize continued reliance on traditional and incremental adaptation strategies, reflecting both local knowledge systems and limited access to formal support mechanisms. These qualitative insights reinforce the interpretation of climate change as a socio-ecological process in which environmental transformations are closely intertwined with economic vulnerability and adaptive responses.

These patterns are further synthesized through both quantitative aggregation and qualitative interpretation of responses, as presented in Tables 4 and 5, allowing for a more integrated understanding of socio-ecological dynamics.

### 3.3 A socio-ecological conceptual framework

Based on both the initial contextual analysis and the empirical findings generated in this study, a socio-ecological conceptual framework was developed (Figure 9).

The framework illustrates the interconnected relationships between climate drivers, environmental transformations, socio-economic impacts, and community responses within a social-ecological system. Climate drivers act as external forcing factors generating biophysical changes, which subsequently affect key livelihood sectors. These impacts are mediated by contextual factors such as institutional access, infrastructure, and livelihood dependence, shaping vulnerability outcomes and potential consequences. ^46^ ^,^ ^53^ ^–^ ^54^

At the same time, communities respond through adaptation strategies, including local knowledge, diversification, and incremental adjustments. The framework integrates both a process-based and systems-oriented perspective, highlighting that these interactions are not strictly linear but are influenced by feedback mechanisms and contextual conditions. Importantly, the relationships represented in the framework reflect perceived socio-ecological dynamics rather than direct causal evidence of climate change impacts.

## 4. Discussion

The statistical results reinforce the existence of a structural gap between climate awareness and response capacity. ^35^ ^,^ ^44^ While the moderate correlation between perceived climate variability and impacts suggests that awareness is closely linked to the recognition of environmental change, the significant differences observed in adaptation and institutional support indicate that this awareness does not necessarily translate into effective response capacity. This divergence indicates the role of structural and institutional factors in shaping adaptation outcomes beyond individual or collective perception.

The findings reveal a clear gap between perception of environmental change and capacity to adapt. Although both community and institutional actors demonstrate high levels of awareness, their ability to respond effectively differs significantly. This gap is not merely a function of knowledge, but rather reflects deeper structural inequalities related to access to resources, institutional support, and technical capacity.

These findings are particularly relevant in Andean highlands socio-ecological systems, where climate-related vulnerabilities are closely associated with territorial isolation, economic dependence on natural resources, and limited institutional presence. In the Lake Titicaca region, adaptive responses are additionally shaped by transboundary governance dynamics between Peru and Bolivia, which may complicate coordinated adaptation efforts. In this context, unequal access to technical assistance, climate information, and financial resources reinforces differentiated adaptive capacities among local actors.

From a socio-ecological perspective, ^46^ these findings show the importance of considering both environmental and social dimensions of vulnerability. Communities are not passive recipients of climate impacts; instead, they actively interpret and respond to environmental change. ^47^ ^–^ ^52^ However, their responses are constrained by broader governance structures that limit access to support mechanisms. This reinforces the notion that adaptation is not solely an individual or local process, but also a systemic one.

The results also support the relevance of socio-ecological resilience frameworks, which emphasize that environmental disturbances and social responses are interconnected through feedback processes operating across multiple scales. In Lake Titicaca communities, local knowledge and incremental adaptation practices function as important resilience mechanisms; however, their effectiveness remains constrained by structural inequalities and fragmented governance systems.

From a policy perspective, ^55^ ^–^ ^56^ the findings underscore the need for integrated approaches to climate adaptation. Strengthening adaptive capacity requires improving access to institutional support, enhancing knowledge exchange, and addressing structural inequalities. Bridging the gap between perception and action is essential for developing effective and equitable climate strategies in high-Andean socio-ecological systems.

Methodologically, the integration of quantitative indicators with participatory and qualitative approaches provided a more comprehensive understanding of climate-related perceptions and adaptive responses. The combination of statistical analysis, participatory mapping, and thematic interpretation allowed the study to capture both measurable patterns and locally grounded experiences of environmental change.

## 5. Conclusions

This study demonstrates that climate change is widely perceived among both community and institutional actors in the Lake Titicaca region. These climate-related perceptions are consistent across different groups and reflect a shared understanding of environmental change. However, climate awareness alone does not guarantee effective adaptation.

The results highlight a significant governance gap in adaptive capacity, whereby communities face greater constraints in responding to climate-related challenges. This gap is shaped by differences in access to resources, institutional support, and knowledge systems. Addressing these disparities is essential for strengthening resilience and reducing vulnerability.

Importantly, the findings should be interpreted as representing perception-based socio-ecological dynamics rather than direct evidence of climatic causality. Nevertheless, the consistency of responses across communities and institutional actors suggests that climate-related environmental transformations are becoming increasingly embedded in local territorial experience and governance challenges. Future research should integrate climatic, hydrological, and longitudinal socio-economic data in order to better understand the interactions between environmental change, institutional adaptation, and community resilience in transboundary high-Andean systems.

Strengthening collaborative and multi-level adaptation strategies will be essential not only for reducing vulnerability, but also for sustaining the ecological integrity and cultural continuity of Lake Titicaca communities under accelerating climate change conditions.

## Data Availability

The following information presents the primary and intermediate processed data that support the results of this article. These data are hosted in the Zenodo repository, which can be freely accessed at Primary data from an article in the journal 1000Research [Data set]. https://doi.org/10.5281/zenodo.18521682. ^57^ Data are available under the terms of the Creative Commons Attribution 4.0 International license (CC-BY 4.0).
