# Supplementary material for: The perception of the impacts of climate change in communities surrounding Lake Titicaca, Peru-Bolivia
Source: F1000Res. 2026 May 26;15:412. Originally published 2026 Mar 19. [Version 2] doi: 10.12688/f1000research.176641.2 (PMC13624497; doi:10.12688/f1000research.176641.2)
Supplement: Peer review history

# Reviewer report: Reviewer response for version 2

**DOI:** 10.5256/f1000research.201536.r488482

**Published:** 2026-09-29

**Authors:** Shamsuddin Shahid

**Affiliations:** 1 National Center for Meteorology, Jeddah, Saudi Arabia

I have carefully reviewed the revised manuscript. The author(s) have satisfactorily addressed all of my comments and concerns. The manuscript has been improved accordingly and is now ready for indexing.

Is the work clearly and accurately presented and does it cite the current literature?

Partly

If applicable, is the statistical analysis and its interpretation appropriate?

No

Are all the source data underlying the results available to ensure full reproducibility?

Partly

Is the study design appropriate and is the work technically sound?

Partly

Are the conclusions drawn adequately supported by the results?

No

Are sufficient details of methods and analysis provided to allow replication by others?

Partly

Reviewer Expertise:

Climate variability and changes, natural hazards

I confirm that I have read this submission and believe that I have an appropriate level of expertise to confirm that it is of an acceptable scientific standard.

# Reviewer report: Reviewer response for version 2

**DOI:** 10.5256/f1000research.201536.r488483

**Published:** 2026-05-28

**Authors:** Jaynos R Cortes

**Affiliations:** 1 North Eastern Mindanao State University- Lianga Campus, Surigao del Sur, Philippines

I have no further comments. The authors have adequately addressed the major concerns raised in the previous review, and the revised manuscript shows substantial improvement in methodological clarity, analytical rigor, and interpretation of results.

Is the work clearly and accurately presented and does it cite the current literature?

Partly

If applicable, is the statistical analysis and its interpretation appropriate?

Partly

Are all the source data underlying the results available to ensure full reproducibility?

Partly

Is the study design appropriate and is the work technically sound?

Yes

Are the conclusions drawn adequately supported by the results?

Yes

Are sufficient details of methods and analysis provided to allow replication by others?

Partly

Reviewer Expertise:

Aquaculture and Fisheries Science; Climate Change Impacts on Aquatic and Fisheries Systems; Fish Nutrition, Health, and Physiology; Community-Based and Participatory Fisheries Research; Sustainable Aquaculture and Fisheries Management; Quantitative and Statistical Analysis in Aquatic Research

I confirm that I have read this submission and believe that I have an appropriate level of expertise to confirm that it is of an acceptable scientific standard.

# Reviewer report: Reviewer response for version 1

**DOI:** 10.5256/f1000research.194720.r469930

**Published:** 2026-04-24

**Authors:** Shamsuddin Shahid

**Affiliations:** 1 National Center for Meteorology, Jeddah, Saudi Arabia

This manuscript addresses an important topic: the multidimensional impacts of climate change on vulnerable transboundary communities surrounding Lake Titicaca. The study focuses on one of South America’s most culturally and hydrologically significant high-altitude lake systems, where climate stress, declining lake levels, pollution, and socio-economic vulnerability interact. The topic is timely, policy-relevant, and internationally important. The good aspect of the paper is its field engagement. The authors conducted participatory workshops, interviews, surveys, institutional meetings, and social mapping in both Peru and Bolivia, which provide local insights that are generally not captured in conventional climate studies.

 However, the manuscript remains methodologically weak in several scientific aspects. The current version is closer to a descriptive field report than a rigorously analyzed research article. My recommendation is a major revision

 Major comments:

 The manuscript states broad objectives but does not clearly define specific objectives. The objective mentioned in the Introduction is also not clear. It seems this article aims to quantify community perceptions of climate change, not to "determine the impacts of climate change on lakeside communities," as mentioned in the Introduction.

 The sampling design is poorly defined. It is mentioned "62 community respondents", "6 interviews", etc. But nowhere is it explained how participants were selected, what type of sampling was used, etc. Also, nothing was mentioned about gender balance and site selection criteria. This is a major methodological weakness.

 Nearly all results are percentages only. For example, "74% observed reduced rainfall", "58% hotter days", etc. But no statistical inference is performed. The article's scientific contribution is very weak.

 One of the major issues is that the author(s) considered the perception as actual climate evidence. For example, they mentioned, “Climate change is causing chronic water deficit…” But survey responses alone cannot establish hydrological causation. Perceptions are important but distinct from measured evidence of climate. Author(s) can use meteorological or hydrological datasets to verify trends. Otherwise, they should rewrite the text.

 For a climate-change paper on Lake Titicaca, it is surprising that no actual climate datasets are analyzed. Even nothing is mentioned about the climate, environment, or hydrology of the region. At least a general description of the region's climate, how it is changing (based on existing literature), and the social and environmental issues are required to understand the background.

  Discussion needs stronger engagement with the literature. Authors can cite existing literature on rainfall trends, temperature trends, lake-level changes, drought changes, etc., to discuss how these findings align with or contradict the community's perception.

 The conclusions are overstated in some cases. Please revise it based on only what you found in your study. Some terms can be revised, such as "chronic water-deficit syndrome", "structural water crisis", and "cascading impacts". You need to be more cautious unless backed by measured data.

 Several grammatical and stylistic issues are present. I hope the author(s) will correct them during revision.

 Minor Comments

 I suggest revising the title. It seems that this article aims to quantify community perceptions of climate change.

 Figure 1: Please improve using a scale bar and coordinates.

 Section 3.1.1.1: Detail description of rituals is not relevant. Please remove/shorten the paragraph.

Is the work clearly and accurately presented and does it cite the current literature?

Partly

If applicable, is the statistical analysis and its interpretation appropriate?

No

Are all the source data underlying the results available to ensure full reproducibility?

Partly

Is the study design appropriate and is the work technically sound?

Partly

Are the conclusions drawn adequately supported by the results?

No

Are sufficient details of methods and analysis provided to allow replication by others?

Partly

Reviewer Expertise:

Climate variability and changes, natural hazards

I confirm that I have read this submission and believe that I have an appropriate level of expertise to confirm that it is of an acceptable scientific standard, however I have significant reservations, as outlined above.

# Response

**Published:** 2026-05-13

**Authors:** Alberto Enrique Garcia Rivero

**Affiliations:** Universidad Nacional Mayor de San Marcos Facultad de Ciencias Sociales, Lima District, Lima Region, Peru

Dear Dr. Shamsuddin Shahid. We greatly appreciate your constructive comments and recommendations provided during the review process. The observations were highly valuable and allowed us to substantially improve the manuscript in terms of methodological rigor, analytical depth, conceptual clarity, statistical treatment, and scientific writing quality.

 In response to your comments, we performed a comprehensive revision of the manuscript. The principal modifications include:

 General assessment: The reviewer considered the topic timely and internationally relevant but stated that the original version was closer to a descriptive field report than to a rigorously analyzed research article.

 A/. The manuscript has been reorganized to strengthen its scientific structure. It now includes a clearer objective, sampling explanation, exploratory statistical analysis, integrated qualitative interpretation, conceptual framework, revised Discussion, and more cautious Conclusions.

 Major Comment 1. Objectives were broad and nuclear: The reviewer noted that the manuscript seemed to quantify community perceptions rather than determine climate-change impacts, and requested clearer specific objectives.

 A/. The research objective was reformulated. The revised manuscript now states that it analyzes climate-change perceptions, perceived impacts, and adaptation capacities among communities and institutional actors in Lake Titicaca. The title and abstract were also adjusted to align the scope with the actual empirical evidence.

 Major Comment 2. Sampling design poorly defined: The reviewer requested explanation of how participants were selected, what type of sampling was used, and information on gender balance and site selection criteria.         

 A/. The Materials and Methods section now explains that the study used purposive, non-probabilistic sampling. Site selection was based on geographic representation, vulnerability/exposure to lake-level and environmental changes, dependence on climate-sensitive activities, and field accessibility. The sample composition is described as 62 community respondents and 26 institutional actors, and the revised text reports gender composition where available. The limitations of the sampling strategy are explicitly acknowledged.

 Major Comment 3. Results were mostly percentages with no statistical inference: The reviewer indicated that the scientific contribution was weak because the original results relied almost exclusively on descriptive percentages.     

 A/. The Results now include comparative indicators, cross-tabulations, composite indices, chi-square tests, and Spearman correlation analysis. The analysis shows significant differences in adaptation measures and institutional support and a moderate correlation between climate perception and reported impacts. These additions substantially increase analytical depth while remaining consistent with the exploratory sample size.

 Major Comment 4. Perception was treated as actual climate evidence: The reviewer warned that survey responses cannot establish hydrological causation and that statements such as “Climate change is causing chronic water deficit” should be rewritten unless supported by measured datasets.

 A/. This was one of the main revisions. The manuscript now clearly distinguishes local and institutional perceptions from measured hydrometeorological evidence. Causal statements were softened or removed. The Discussion and conceptual framework explicitly state that the study examines perceived socio-ecological dynamics rather than direct causal evidence. The term “chronic water-deficit syndrome” and similar overstated language were removed from the Conclusions.

 Major Comment 5. Lack of climate, environmental, and hydrological background: The reviewer requested at least a general description of regional climate, environmental issues, hydrology, and existing evidence of change.      

 A/. The Introduction and Discussion were expanded to situate the study within the high-Andean socio-ecological context of Lake Titicaca. The revised text now discusses climate variability, hydrological variability, lake-level decline, pollution pressures, livelihood dependence, and transboundary governance as contextual factors. This background is based on literature rather than new climate-data analysis.

 Major Comment 6. Discussion needed stronger engagement with literatura: The reviewer requested comparison with existing literature on rainfall, temperature, lake-level changes, drought, and adaptation.

 A/. The Discussion was substantially rewritten. It now connects the perception-based results with literature on socio-ecological systems, climate vulnerability, local adaptation, participatory climate research, hydrological variability, and Lake Titicaca-related environmental stress. The revised text also explains where findings align with broader research and where they should be interpreted cautiously because direct climate datasets were not analyzed.

 Major Comment 7. Conclusions overstated:  The reviewer recommended revising terms such as “chronic water-deficit syndrome,” “structural water crisis,” and “cascading impacts,” and ensuring the Conclusions are based only on the study findings. 

 A/. The Conclusions were rewritten to focus only on findings supported by the survey, interview, participatory, and exploratory statistical evidence. The revised Conclusions emphasize high perceived climate awareness, uneven adaptive capacity, disparities in institutional support, and the need for multi-level governance, participatory adaptation, and transboundary cooperation.

 Major Comment 8. Grammar and style: The reviewer noted several grammatical and stylistic issues.  

 A/. The revised manuscript underwent language editing to improve academic style, reduce excessive descriptive wording, and make the structure more concise and coherent.

 Minor Comment: revise title: The reviewer suggested revising the title because the article primarily quantifies perceptions.             

 A/. The title was revised to foreground perception: “The perception of the impacts of climate change in communities surrounding Lake Titicaca, Peru-Bolivia.”

 Minor Comment: Figure 1: The reviewer requested improving Figure 1 with scale bar and coordinates.      

 A/. The location figure was revised/clarified and its caption improved. To avoid overburdening the map visually, the revision prioritized clearer location interpretation and captioning. If the journal requests, a final cartographic version with scale bar and coordinates can be supplied as a figure file.

 Minor Comment: rituals paragraph: The reviewer suggested removing or shortening the detailed ritual description in Section 3.1.1.1.     

 A/. The ritual description was shortened and repositioned as contextual qualitative evidence related to traditional knowledge and adaptation practices, avoiding excessive ethnographic detail that was not central to the research objective.

# Reviewer report: Reviewer response for version 1

**DOI:** 10.5256/f1000research.194720.r471132

**Published:** 2026-04-01

**Authors:** Jaynos R Cortes

**Affiliations:** 1 North Eastern Mindanao State University- Lianga Campus, Surigao del Sur, Philippines

**General Assessment**

 This manuscript presents a multidisciplinary and participatory assessment of climate change impacts on lakeside communities surrounding Lake Titicaca. The integration of document review, field surveys, interviews, and participatory mapping is a notable strength and provides valuable socio-ecological insights. The topic is highly relevant, particularly in the context of climate vulnerability, transboundary water management, and community-based adaptation.

 However, despite its relevance and strong empirical foundation, the manuscript requires **substantial improvements in methodological rigor, analytical depth, and clarity of data interpretation** to meet the standards of scientific validity.

**Major Comments**

**1. Methodological Rigor and Sampling Design**

 While the study employs multiple methods, the **sampling framework lacks sufficient detail and justification**. Specifically:

- The basis for site selection (Quehuaya, Capachica, Guaqui, and Puno) should be clarified (e.g., representativeness, vulnerability criteria).
- Sample size (e.g., n = 26 for seminar survey; n = 62 community respondents) is relatively limited and requires justification regarding statistical adequacy.
- The study lacks a discussion of potential sampling bias (e.g., purposive vs random sampling).

Recommendation: Include a clear **sampling design subsection** and justify representativeness and limitations.

**2. Data Analysis and Statistical Treatment**

 The manuscript states that data were “synthesized quantitatively,” but:

- No statistical tests (e.g., regression, correlation, comparative analysis) are presented.
- Results rely heavily on descriptive percentages without inferential support.
- Relationships between variables (e.g., climate perception vs livelihood impacts) are not explored.

Recommendation: Incorporate:

- Basic inferential statistics (e.g., chi-square, correlation analysis)
- Cross-tabulation of key variables
- Clear analytical framework linking variables

 

**3. Integration of Qualitative and Quantitative Data**

 Although the study uses mixed methods, the **integration remains descriptive rather than analytical**:

- Interview findings are presented narratively but not systematically coded
- Participatory mapping outputs are described but not quantitatively or spatially analyzed

Recommendation: Apply:

- **Thematic analysis** for qualitative data
- Structured interpretation linking qualitative insights with survey results

 

**4. Conceptual and Theoretical Framework**

 The manuscript lacks a **clear conceptual framework**:

- No explicit linkage between climate change drivers, socio-economic vulnerability, and adaptation responses
- Limited engagement with theoretical models (e.g., socio-ecological systems, resilience theory)

Recommendation: Include a **conceptual model** to guide interpretation and strengthen analytical coherence.

**5. Interpretation of Results and Causality**

 Several conclusions appear **overgeneralized or insufficiently supported**:

- The concept of “chronic water-deficit syndrome” is introduced without clear operational definition
- Causal relationships between climate change and socio-economic outcomes are implied rather than demonstrated

Recommendation:

- Clearly define key terms
- Avoid causal claims unless supported by data
- Strengthen linkage between results and conclusions

 

**6. Limited Fisheries-Specific Analysis**

 Although fisheries are mentioned as impacted (e.g., 52% decline in fish availability), the analysis:

- Lacks species-level data
- Does not quantify fishing effort or ecological changes

Recommendation:

 Expand the fisheries analysis or clarify its limitations as perception-based data.

**Minor Comments**

- Improve clarity and conciseness of long descriptive paragraphs
- Ensure consistency in reporting percentages and units
- Figures (e.g., participatory maps) require clearer interpretation and captions
- Some references are outdated or overly generalized—include more recent region-specific studies

 

**CLARIFICATION ON REFERENCES**

 The references included, were intended to support improvements in:

- **Methodology section** → for strengthening mixed-methods and participatory approaches
- **Discussion section** → for situating findings within broader climate vulnerability and socio-ecological literature

Specifically:

- Suggested references should be incorporated in the **Discussion** to support claims on:
  - Climate vulnerability
  - Community adaptation
  - Socio-ecological systems

 

**FINAL CONFIRMATION**

**✔** **Recommended Decision: APPROVED WITH RESERVATIONS**

 The manuscript addresses an important and timely topic and demonstrates strong field engagement. However, **moderate to major revisions** are required to improve methodological rigor, analytical depth, and scientific clarity before it can be considered fully valid.

Is the work clearly and accurately presented and does it cite the current literature?

Partly

If applicable, is the statistical analysis and its interpretation appropriate?

Partly

Are all the source data underlying the results available to ensure full reproducibility?

Partly

Is the study design appropriate and is the work technically sound?

Yes

Are the conclusions drawn adequately supported by the results?

Yes

Are sufficient details of methods and analysis provided to allow replication by others?

Partly

Reviewer Expertise:

Aquaculture and Fisheries Science; Climate Change Impacts on Aquatic and Fisheries Systems; Fish Nutrition, Health, and Physiology; Community-Based and Participatory Fisheries Research; Sustainable Aquaculture and Fisheries Management; Quantitative and Statistical Analysis in Aquatic Research

I confirm that I have read this submission and believe that I have an appropriate level of expertise to confirm that it is of an acceptable scientific standard, however I have significant reservations, as outlined above.

## References

[1] : Resilience: The emergence of a perspective for social–ecological systems analyses. *Global Environmental Change* .2006;16(3) : 10.1016/j.gloenvcha.2006.04.002253-267 10.1016/j.gloenvcha.2006.04.002

[2] : Participatory approaches to climate adaptation, resilience, and mitigation: A systematic review. *Ambio* .2025;54(12) : 10.1007/s13280-025-02202-z2005-2020 10.1007/s13280-025-02202-z PMC1256930340465160

[3] : Untangling the effects of climate variability and irrigation management on water levels in Lakes Titicaca and Poopó. *Journal of Hydrology: Regional Studies* .2021;37: 10.1016/j.ejrh.2021.100927 10.1016/j.ejrh.2021.100927

# Response

**Published:** 2026-05-13

**Authors:** Alberto Enrique Garcia Rivero

**Affiliations:** Universidad Nacional Mayor de San Marcos Facultad de Ciencias Sociales, Lima District, Lima Region, Peru

Dear reviwer Dr. Jaynos Cortes. We greatly appreciate your constructive comments and recommendations provided during the review process. The observations were highly valuable and allowed us to substantially improve the manuscript in terms of methodological rigor, analytical depth, conceptual clarity, statistical treatment, and scientific writing quality.

 In response to your comments, we performed a comprehensive revision of the manuscript. The principal modifications include:

 General assessment: The reviewer recognized the relevance of the topic and the value of the multidisciplinary and participatory approach but indicated that the manuscript required substantial improvements in methodological rigor, analytical depth, and clarity of data interpretation.  

 A/. The manuscript was substantially revised. The scope was clarified as a perception-based mixed-methods study; methodological transparency was expanded; inferential exploratory analyses were added; the qualitative component was reorganized; and the Discussion and Conclusions were rewritten to avoid unsupported causal claims.

 Major Comment 1. Methodological rigor and sampling design:The reviewer requested clarification of the basis for site selection, justification of the limited sample size, and discussion of potential sampling bias, including purposive versus random sampling.

 A/. A sampling-design explanation was added in Materials and Methods. The revised text states that the strategy was purposive and non-probabilistic and that sites were selected according to geographic representation, exposure/vulnerability to lake changes, dependence on climate-sensitive livelihoods, and accessibility. The manuscript now reports the final sample structure: 62 community respondents from Quehuaya and Capachica and 26 institutional actors associated with tourism and environmental management. A limitation statement was added indicating that the sample is not statistically representative of the entire Lake Titicaca basin and is best interpreted as exploratory evidence for socio-ecological analysis and hypothesis generation.

 Major Comment 2. Data analysis and statistical treatment: The reviewer noted that the original version relied mostly on descriptive percentages and did not include tests, correlations, comparative analyses, or exploration of relationships between variables such as climate perception and livelihood impacts.         

 A/. The revised manuscript now includes an analytical framework based on binary variables, composite indices, cross-tabulations, chi-square tests, and Spearman correlations. The new analysis identifies significant differences between communities and institutional actors in adaptation measures (χ² = 14.96, p < 0.001) and institutional support (χ² = 18.32, p < 0.001). It also reports a moderate positive association between climate perception and reported impacts (ρ = 0.36, p = 0.003). These results are interpreted cautiously as exploratory associations, not causal relationships.

 Major Comment 3. Integration of qualitative and quantitative data         .The reviewer indicated that the mixed-methods integration remained mostly descriptive and recommended thematic analysis and structured interpretation linking interviews and participatory mapping with survey findings.

 A/. The qualitative evidence was reorganized through thematic coding and socio-ecological interpretation. The manuscript now identifies recurrent qualitative categories, including water stress, livelihood impacts, adaptation practices, environmental change, shoreline retreat, and institutional support limitations. These themes are explicitly linked to survey results and statistical findings, especially the gap between high climate awareness and uneven adaptive capacity. Participatory mapping is now described as contextual and interpretive evidence rather than as a complete spatial quantification.

 Major Comment 4. Conceptual and theoretical framework: The reviewer requested a clearer framework linking climate drivers, socio-economic vulnerability, and adaptation responses, with engagement with socio-ecological systems and resilience theory.

 A/. A socio-ecological conceptual framework was added. The new framework links climate drivers, environmental transformations, socio-economic impacts, contextual vulnerability factors, and community/institutional adaptive responses. The Introduction and Discussion were strengthened with socio-ecological systems and resilience literature, including the reviewer’s recommended line of interpretation. The framework clarifies that environmental change and social response are connected through feedbacks and mediating factors such as institutional access, technical knowledge, resources, infrastructure, and livelihood dependence.

 Major Comment 5. Interpretation of results and causality:           The reviewer warned that some conclusions were overgeneralized, that the term “chronic water-deficit syndrome” was insufficiently defined, and that causal relationships between climate change and socio-economic outcomes were implied rather than demonstrated.    

 A/. Overstated causal language was removed or replaced with more cautious wording. The revised manuscript explicitly states that the relationships analyzed are perception-based and should not be interpreted as direct hydrometeorological causation. The problematic phrase “chronic water-deficit syndrome” was removed from the revised conclusion. The Conclusions now state that climate change is widely recognized by respondents and that adaptive capacity is uneven due to disparities in institutional support, resources, and technical knowledge.

 Major Comment 6. Limited fisheries-specific analysis: The reviewer noted that fisheries were mentioned as impacted but that the study did not include ecological measurements, fishing effort data, or quantified species-level analysis.

 A/. The fisheries section was revised to make clear that species-level references are based on community perception and reported experience, not on independent biological monitoring. The manuscript retains reported references to species such as carachi, pejerrey, umanto, ispi, suche, and boga because they are important local evidence, but it now frames them as perceived declines in availability or catch rather than quantified ecological trends. The limitation that no direct fisheries effort or ecological survey was conducted is acknowledged.

 Minor Comment: long descriptive paragraphs: The reviewer requested improved clarity and conciseness.      

 Several long descriptive passages were shortened or reorganized. The Results were divided into clearer analytical subsections, and the Discussion was rewritten to avoid repetition and improve scientific flow.

 Minor Comment: consistency in percentages and units: The reviewer requested consistent reporting of percentages and units.

 A/. Percentages and sample descriptors were checked and standardized. The revised manuscript reports values consistently, including community respondents, institutional actors, and comparative indicators.

 Minor Comment: figures and captions: The reviewer requested clearer interpretation and captions for figures, including participatory maps.          

 A/. Figure captions were revised to better indicate what each figure shows and how it should be interpreted. Participatory figures are now treated as qualitative visual evidence generated through community mapping, not as exhaustive cartographic measurement.

 Minor Comment: references: The reviewer recommended adding recent and region-specific studies and clarified that suggested references should support the Methods and Discussion.

 A/. The manuscript incorporated additional literature in the Methods and Discussion to support mixed-methods design, participatory approaches, socio-ecological systems, vulnerability, adaptation, resilience, and Lake Titicaca-related hydrological/environmental change.
